# Heterogeneous donor circles for fair liver transplant allocation

**DOI:** 10.1007/s10729-022-09602-7

**Published:** 2022-07-20

**Authors:** Shubham Akshat, Sommer E. Gentry, S. Raghavan

**Affiliations:** 1 The Robert H. Smith School of Business, University of Maryland, College Park, MD 20742 USA; 2 Department of Surgery and Department of Population Health, Grossman School of Medicine, New York University, New York, NY 10016 USA; 3 The Robert H. Smith School of Business and Institute for Systems Research, University of Maryland, College Park, MD 20742 USA

**Keywords:** Health care policy, Liver transplant, Geographical disparity, Optimization, Operations research

## Abstract

The United States (U.S.) Department of Health and Human Services is interested in increasing geographical equity in access to liver transplant. The geographical disparity in the U.S. is fundamentally an outcome of variation in the organ supply to patient demand (s/d) ratios across the country (which cannot be treated as a single unit due to its size). To design a fairer system, we develop a nonlinear integer programming model that allocates the organ supply in order to maximize the minimum s/d ratios across all transplant centers. We design circular donation regions that are able to address the issues raised in legal challenges to earlier organ distribution frameworks. This allows us to reformulate our model as a set-partitioning problem. Our policy can be viewed as a heterogeneous donor circle policy, where the integer program optimizes the radius of the circle around each donation location. Compared to the current policy, which has fixed radius circles around donation locations, the heterogeneous donor circle policy greatly improves both the worst s/d ratio and the range between the maximum and minimum s/d ratios. We found that with the fixed radius policy of 500 nautical miles (NM), the s/d ratio ranges from 0.37 to 0.84 at transplant centers, while with the heterogeneous circle policy capped at a maximum radius of 500 NM, the s/d ratio ranges from 0.55 to 0.60, closely matching the national s/d ratio average of 0.5983. Our model matches the supply and demand in a more equitable fashion than existing policies and has a significant potential to improve the liver transplantation landscape.

## Highlights

- Problem specification: This paper focuses on improving geographic equity in access to liver transplant in the United States (U.S.). Prior organ distribution policies have resulted in great disparities in the organ supply to candidate demand (s/d) ratios across transplant centers, causing large differences in waiting times and mortality rates across them.
- Core insight: A fixed radius donor circle policy, where transplant centers within a fixed distance from a donor hospital get priority access to livers, does not reduce the variation in the s/d ratio across transplant centers. Rather, a customized “heterogeneous” approach that accounts for both the organ supply and candidate demand locations and adjusts the radii of the donor circles more effectively addresses geographic equity by equalizing the s/d ratios across all transplant centers to closely match the national s/d ratio.
- Practical implications: Explicitly accounting for the local variation in the organ supply and candidate demand can lead to further improvements in organ distribution policy—which will ultimately result in robust policies promoting greater equity for transplant candidates across the U.S.

## Introduction

**Fig. 1:**
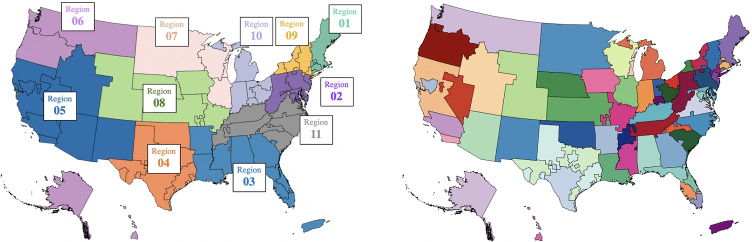
Pre February 4, 2020 policy divided the U.S. into 11 regions (left), which comprised 58 DSAs (right)

In 2019, 8,896 liver transplants took place in the United States (U.S.), while 12,941 patients were added to the waiting list. Unfortunately, in 2019, on average three people in the U.S. died every day awaiting a liver transplant, for a total of 1,202 lives lost. Because demand for liver transplant outstrips supply, allocating deceased donor livers judiciously and justly is extremely important. For 30 years (from 1989 to Feb. 4, 2020) the transplant allocation policy divided the U.S. into 58 Donation Service Areas (DSAs), which were grouped into 11 geographical regions (Fig. 1). Livers were offered to candidates in a DSA in decreasing order of medical urgency, quantitatively measured by the Model for End-stage Liver Disease (MELD) score (the Pediatric End-Stage Liver Disease (PELD) severity score, a measure calculated slightly differently, is used for patients ≤ 12 years old). The MELD score reflects the probability of death within a three-month period and ranges from 6 to 40; a higher score indicates a greater mortality risk [10]. More serious patients are assigned Status 1A and 1B, and their number is fewer than 50 nationwide at any time.

By law, the deceased donor organs are national resources in the U.S. The U.S. government created the Organ Procurement and Transplantation Network (OPTN) in 1984 to coordinate a nationwide transplant system and optimize the usage of the limited resource of donor organs for transplants. Since 1986, the United Network for Organ Sharing (UNOS), a nonprofit private organization, has overseen OPTN operations. A key regulatory framework guiding organ transplantation is the “Final Rule,” which was adopted in 1998 by the Department of Health and Human Services (HHS) to establish a more detailed framework for OPTN’s structure and operations [19]. The Final Rule *requires that policies shall not be based on the candidate’s place of residence or place of listing, except to the extent required by the other conditions of the Rule*.

Geographic inequity in access to liver transplantation across DSAs is well documented in the literature [32]. Indeed, as early as 2008, an HHS Advisory Committee on Transplantation recommended that organ allocation be evidence-based and not on the arbitrary boundaries of the DSAs. In 2012, the OPTN board adopted a strategic plan that included reducing geographic disparities in access to transplantation. Despite implementation in 2013 of broader organ sharing in a region for candidates with MELD scores ≥ 35, geographic inequities remained in the system. The U.S. Scientific Registry of Transplant Recipients’ (SRTR’s) Liver Transplant Waiting List Outcomes Tool^1^ (built on historical data from 2017 to 2019) shows that for waitlisted candidates in Los Angeles with MELD scores in the range of 25-29, only 15% received a transplant within 90 days, while for candidates in Indianapolis (with MELD scores in the range of 25-29), 72% received a transplant within 90 days. The DSA/Region allocation policy resulted in significant disparities even for candidates on transplant lists in close proximity. For example, SRTR’s Liver Transplant Waiting List Outcomes Tool shows that for waitlisted candidates in New York City with MELD scores in the range of 25-29, only 15% received a transplant within 90 days, while for similar candidates in Newark, New Jersey, just 15 miles away, 41% received a transplant within 90 days. Because MELD scores directly correlate with the probability of death in the absence of an organ transplant in the next 90 days, different transplant wait times for candidates with the same MELD score across DSAs imply (i) significantly different mortality rates for candidates with the same MELD score in different DSAs, and (ii) significant variation in the median MELD score at transplant (MMaT).^2^ Indeed, MMaT variance has typically been used by UNOS as a key metric in evaluating a proposal’s effectiveness in mitigating geographic disparity (i.e., a lower value of MMaT variance indicates less disparity).

In November 2017, New York City resident Miriam Holman (a patient with a rare form of pulmonary hypertension for which there is no medical therapy, and which is rapidly fatal without lung transplantation) filed a lawsuit (hereafter, “lung lawsuit”) against HHS.^3^ Due to the particular lung allocation policy in place at that time, a donor lung could become available across the river in New Jersey (less than four miles away). However, because the location of the donor lung was in a different geographical DSA, it had to be offered to every candidate waiting for lungs in that New Jersey DSA (even to candidates who were much farther away and far less medically critical) before it could be offered to Holman [14]. In July 2018, six liver transplant waiting list patients in New York, California, and Massachusetts filed a lawsuit (hereafter, “liver lawsuit”) against HHS.^4^ The liver lawsuit pointed out the wide geographical variability in the median MELD scores in recipients for deceased donor transplants, arguing that the place of residence largely determines the chances of one’s survival in the existing policy.

To address these issues, in June 2018, the UNOS board (based on the recommendations of a Geography Committee formed in December 2017) adopted the following set of principles to guide future organ transplant policy relating to the geographic aspects of organ distribution (which were also identified as being consistent with the final rule).

1. Reduce inherent differences in the ratio of the donor supply and demand across the country.
2. Reduce travel time expected to have a clinically significant effect on ischemic time and organ quality.
3. Increase organ utilization and prevent organ wastage.
4. Increase efficiencies of donation and transplant system resources.

The Geography Committee identified three potential distribution frameworks that fit with these four principles: (1) fixed distance from the donor hospital, (2) mathematically optimized boundaries, and (3) continuous scoring (candidates to be ranked on the offer list on a combination of their clinical characteristics and proximity to a donor).^5^

Following public comment, on December 3, 2018, the UNOS board adopted an Acuity Circle policy (an implementation of the fixed distance from the donor hospital framework that we discuss in Section 2.2). Although there were legal challenges and political pressures from several quarters to maintain the existing system, the new Acuity Circle policy was implemented on May 14, 2019. However, within a day, on May 15, 2019, a federal court issued an injunction, and UNOS was required to revert to the prior system while legal challenges to the policy were pending. On January 16, 2020, the federal court reversed itself and decided not to keep the injunction in place while the case was pending. Subsequently, the Acuity Circle policy was once again implemented again on February 4, 2020.

In this paper, we use UNOS’s stated principle of reducing inherent differences in the ratio of the supply to demand (s/d) as our objective explicitly within a mathematical optimization framework to design heterogeneous sized areas around the donation locations. One approach to reduce inequity is through the central distributive principle, proposed by Rawls [26]: the least well-off group in the society should be made as well off as possible. We use this *maximin* principle to design heterogeneous sized areas that maximize the minimum value of the s/d ratio across all transplant centers (or DSAs). We then apply a secondary optimization to minimize the disparity between the transplant centers (or DSAs) with the highest and lowest s/d ratios.

Our mathematical optimization model can be applied using zip codes or DSAs as the geographical units. When using zip codes as the geographical units, the model may be viewed as a heterogeneous radii circle policy (as compared to a fixed radius circle policy^6^). When using DSAs as the geographical units, the model may be viewed as a type of neighborhood model [20], where the neighborhood around a DSA is somewhat circular in shape.

Without organ sharing among DSAs, we found that the s/d ratio ranges from 0.31 to 1.98. With 500 nautical-mile (NM) fixed circles, the s/d ratio improves and ranges from 0.37 to 0.84. We show that when heterogeneous circles are used around the donation zip codes, the s/d ratio ranges from 0.55 to 0.60, meaning that there is a much lower disparity in organ access among the transplant centers. Further, when we examine the s/d ratio disparity for transplant centers that are close to one another (specifically, within 150 NM of each other) the heterogeneous circle policy reduces the s/d ratio disparity to one-fourth compared to the fixed 500 NM circle policy.

We ran simulations with SRTR’s Liver Simulated Allocation Model (LSAM, version 2014) using historical patient and organ donor data. The version of the tool available to us was based on DSAs. Hence, we compared our optimized geographical neighborhoods using DSAs. The results show that in comparison to the prior OPTN 11 region policy (in place until February 4, 2020), an allocation policy based on our optimized heterogeneous circular neighborhoods (around DSAs), with a maximum radius of 500 NM and full regional sharing of all organs with MELD scores ≥ 15, drastically reduces the variance of MMaT across DSAs (from 13.66 to 2.00) and average annual deaths (from 3,745 to 3,568), for a modest increase in average travel distance (from 199 NM to 258 NM).

A key policy insight is that the one-size-fits-all framework (i.e., the currently proposed Acuity Circle policy) approach taken by UNOS does not adequately address the problem of reducing differences in the ratio of the donor supply to demand across the country. Rather, a customized approach that accounts for where the organ supply and demand occur and adjusts radii of the circles more effectively addresses UNOS’ stated goal of equalizing s/d ratios. The remainder of the paper is organized as follows. In the next section, we give a brief overview of the liver allocation system in the U.S., and review proposals and related research. Section 3 presents our optimization methodology. Section 4 describes our findings and projected outcomes. Section 5 summarizes and provides concluding remarks.

## Liver allocation policy and literature review

UNOS supervises the transplantation network in the U.S. Its primary responsibilities are to manage the national transplant waiting list, match organs from deceased donors to candidates, establish medical criteria for allocating organs, facilitate organ distribution, frame equitable policies, etc. Some of the main UNOS members are the 142 liver transplant centers and Organ Procurement Organizations (OPOs) in the 58 DSAs. The OPO coordinates the local procurement of deceased donor organs and allocation in a DSA.

Each transplant center evaluates patients and adds candidates to the waitlist. The medical data about the candidates are shared with UNOS. These pooled data of candidates across all transplant hospitals are constantly updated when new candidates get added, and existing candidates are either removed or their medical conditions (e.g., MELD scores) are updated. When a deceased donor organ becomes available, the OPO sends medical data about the organ donor to UNOS. Subsequently, the UNOS matching system compares the donor information with the candidate pool to rank candidates for the organ offer as per the allocation policy. Upon receiving an offer, the transplant surgeon or physician, in consultation with the candidate, decides whether to accept the offer.

### Share 35 policy

In the previous allocation policy (Share 35) in place from June 18, 2013, until February 4, 2020, deceased donor livers were offered hierarchically to candidates in decreasing order of the MELD scores within each hierarchy, according to the priority list in Table 1. First, an organ was offered to Status 1A and 1B candidates in the region, followed by candidates with MELD scores ≥ 35. After that, candidates with MELD scores between 15 and 35 in the OPO’s DSA were preferred over candidates outside the DSA. Next in the hierarchy were candidates with MELD scores between 15 and 35 in the OPO’s region, followed by candidates with MELD scores between 15 and 35 outside the OPO’s region. Finally, candidates with MELD scores ≤ 15 in the DSA were preferred over candidates with MELD scores ≤ 15 outside the DSA but in the region, who in turn were preferred over candidates with MELD scores ≤ 15 outside the region.

**Table 1:** Liver Allocation Policy (Share 35) prior to February 4, 2020

Sequence #	Candidates that are within:	And are:
1	OPO’s region	Adult status 1A or pediatric status 1A/1B
2	OPO’s region	MELD/PELD ≥ 35
3	OPO’s DSA	MELD/PELD ≥ 15
4	OPO’s region	MELD/PELD ≥ 15
5	Nation	Adult status 1A or pediatric status 1A/1B
6	Nation	MELD/PELD ≥ 15
7	OPO’s DSA	MELD/PELD ≤ 15
8	OPO’s region	MELD/PELD ≤ 15
9	Nation	MELD/PELD ≤ 15

**Fig. 2:**
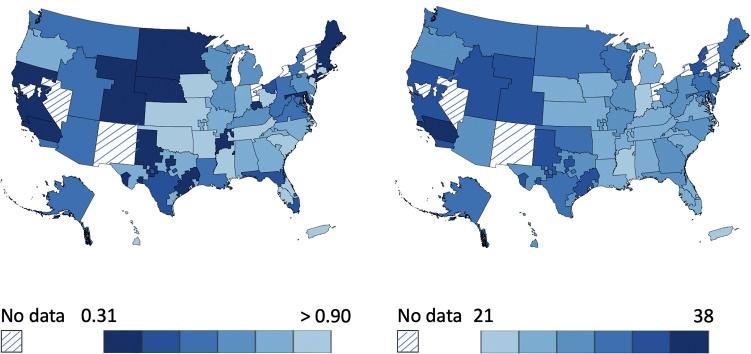
Lower supply to demand (s/d) ratios at a DSA (left) correspond to a higher MMaT at the DSA (right). The time period of analysis is from July, 2013 to June, 2017

Due to differences in demographics, disease incidence, and mortality leading to organ donations among the DSAs, there was a huge disparity in the s/d ratios across the DSAs. Figure 2 shows the wide variability in the s/d ratio (left) and an inverse relationship of this variability with observed MMaT scores (right). The s/d ratios (at DSAs) varied from 0.31 in NYRT (a DSA in New York) to 1.98 in FLWC (a DSA in Florida). This disparity primarily drove the differences in MMaT among the DSAs. In a study by Wey et al. [31], the s/d ratios in a DSA were found to be associated with MMaT in DSAs (*r* = − 0.56;*P* < 0.001).

### Current policy: acuity circles

This policy progressively shares organs in circles of radii 150 NM, 250 NM, and 500 NM around the donor hospital, with the following hierarchy: (1) Status 1 candidates within 500 NM; (2) candidates with MELD scores ≥ 37 within 150 NM, then 250 NM, then 500 NM; (3) candidates with MELD scores ≥ 33 within 150 NM, then 250 NM, then 500 NM; (4) candidates with MELD scores ≥ 29 within 150 NM, then 250 NM, then 500 NM; (5) candidates with MELD scores ≥ 15 within 150 NM, then 250 NM, then 500 NM, and then nationally.^7^ This is a “one-size-fits-all” policy because it does not account for the organ arrival rate, the candidate waiting list, or the distances of the transplant centers from a donor hospital.

### Related research

Redistricting is a problem that occurs frequently in multiple domains (e.g., political redistricting, school redistricting, and sales territory assignments) where a finite, denumerable set of non-overlapping geographical units are aggregated into regions/districts subject to some criteria. Hess et al. [18] and Garfinkel and Nemhauser [11] introduced the use of optimization techniques for political redistricting. Zoltners and Sinha [33] discuss an application of redistricting in sales territory assignments, and Caro et al. [6] discuss school redistricting using integer programming. Much of the redistricting literature focuses on political redistricting (see [15, 21, 27]). Two important considerations in redistricting problems are the contiguity and compactness of the districts. In this regard, Shirabe [28] proposed a flow-based model for contiguity constraints, which has been typically used in subsequent integer programming approaches. However, contiguity constraints make redistricting problems notoriously hard to solve exactly (see [21, 27]).

Focusing on transplants, and disregarding geographical equity for the moment, Kong et al. [23] studied the problem of maximizing efficiency by maximizing total intraregional transplants by redesigning of the liver allocation regions. They formulate the problem as a set-partitioning problem and use a branch-and-price algorithm to approximate solutions. Stahl et al. [30] consider geographical equity as measured using intraregional transplant rates in their objective function along with efficiency (measured by total intraregional transplants), but they restrict their regions to contain up to eight DSAs due to computational challenges. Extending their work, Demirci et al. [7] developed a branch-and-price algorithm to incorporate a larger set of potential regions and explored the efficient frontier in a trade-off between efficiency and geographical equity. Their metric of geographical equity maximizes the minimum in-district viability-adjusted transplant rates per waiting list candidate, which is sensitive to the number of waiting list patients added by the transplant centers, and thus, is problematic. For low-MELD patients, the survival benefit of transplantation is minimal [24], and the chances of receiving an organ vary across geographies. Consequently, the transplant centers differ in their practices of adding low-MELD patients to the waiting list.

Gentry et al. [13] used optimization to reorganize DSAs into regions/districts to reduce geographical disparity. Their objective was to minimize the sum of the absolute differences between the number of deceased-donor livers recovered in each district and the ideal number of livers that would be offered in each district if each liver was given to the medically most urgent candidate in the country. Working closely with the liver committee of UNOS, they proposed eight-district and four-district (reorganized DSA) maps. The proposed maps were under active consideration by UNOS from 2015 to 2017. However, ultimately after significant debate and public comment, they were not adopted.

**Fig. 3:**
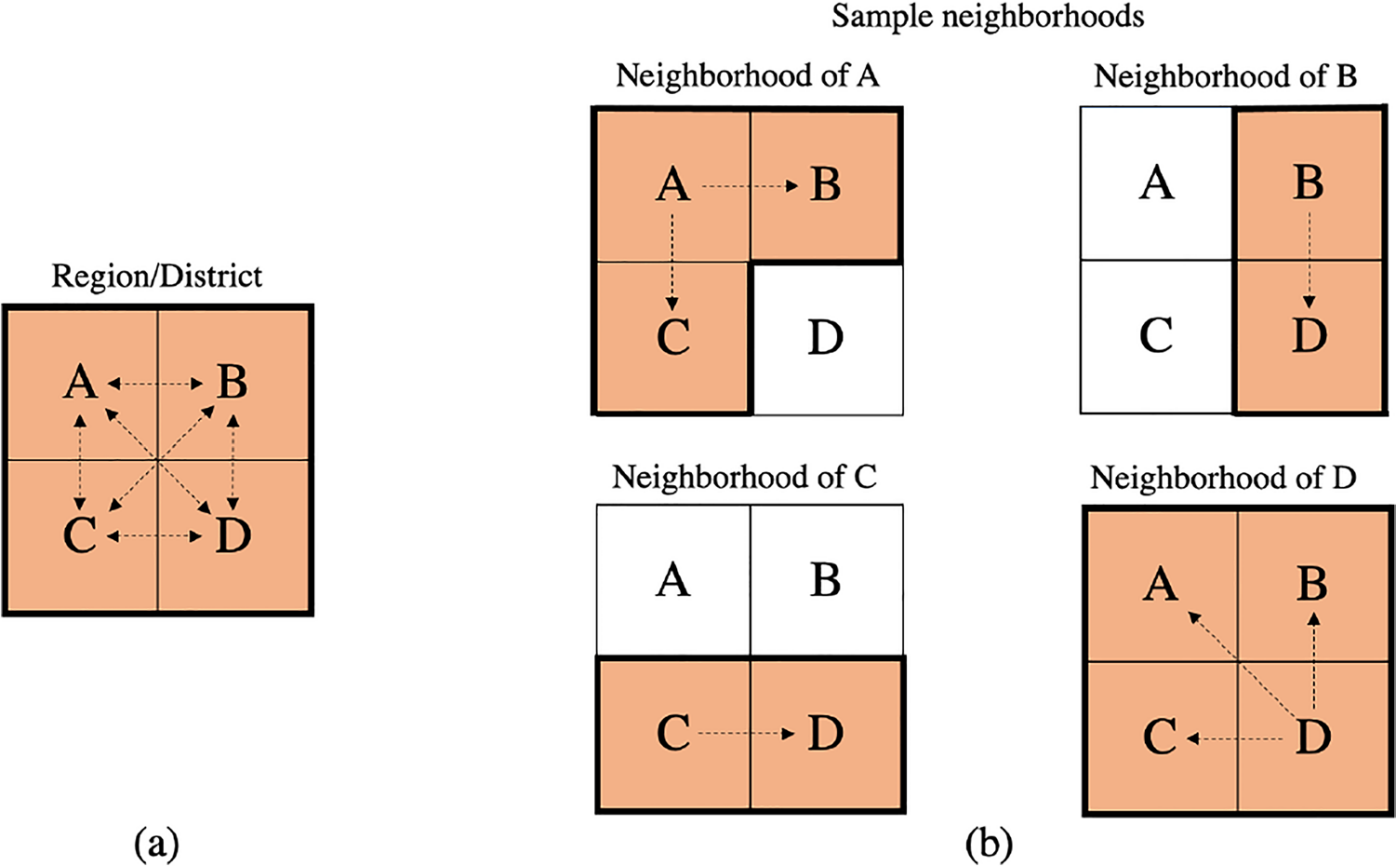
Illustration of the difference between Regions/Districts and neighborhoods. (**a**) Let DSAs A, B, C and D form a region or district. They all share with each other. (**b**) With the neighborhood displayed, the neighborhood of A consists of DSAs A, B and C. Therefore, A shares only with A, B, and C. Similarly, B shares with B and D; C shares with C and D; and D shares with A, B, C, and D

Kilambi and Mehrotra [20] introduced the neighborhood framework in organ allocation as a way to provide for broader sharing and improve geographic equity. Each DSA has its own neighborhood, which consists of a unique set of other DSAs (or neighbors) with which it shares its organs. A DSA can be part of multiple neighborhoods; therefore, the neighborhoods can be overlapping, which makes it difficult to represent all neighborhoods on a single map. Interconnectivity and overlap among neighborhoods provide resilience to supply and demand uncertainty. The neighborhood framework reduces to redistricting when all the DSAs in a neighborhood have the identical neighborhood. Thus, the redistricting framework can be viewed as a special case of a neighborhood framework. Figure 3 illustrates the difference between regions/districts and the neighborhood framework. Using the neighborhood framework, Kilambi and Mehrotra [20] developed an optimization model to design DSA neighborhoods to minimize the absolute deviation of the s/d ratios across neighborhoods from the national average.

Ata et al. [4] used fluid approximation and game theory to show that multiple listings (a patient is listed at more than one transplant center, potentially in another DSA or region so that he/she can get organ offers from multiple places) can reduce geographical disparity in kidney allocation. However, fewer than 2% of patients waiting for a liver transplant multiple list (on April 14, 2021, the OPTN website shows that only 181 out of 11868 candidates are multiple listed). Bertsimas et al. [5] suggest the use of tradeoff curves to assess the three organ distribution frameworks identified by the Geography Committee. Running a large number of simulations for the three distribution frameworks, they plot tradeoff curves of efficiency (measured as average travel distance) versus fairness (measured as deaths or variance of MMaT). For a given value of the efficiency metric, the tradeoff curve then identifies the policy with the greatest fairness. However, they did not consider the neighborhood or heterogeneous circle distribution frameworks in their study. In a recent study, Ata et al. [3] analyze a broad class of ranking policies in kidney allocation using an analytical framework. They find that allowing different patients’ ranking rules, depending on the quality of the kidney, can reduce organ wastage.

There are two methods of organ donation: (1) living donation and (2) deceased donation. Alagoz et al. [2] study the optimal timing of living-donor liver transplantation when the patient is either ineligible or has decided not to receive organs from deceased donors. They ignore the risk to living donors in their model. Ergin et al. [8] model liver exchange as a market-design problem, where they account for risk to donors and compatibility issues. Using data from South Korea, they show that their proposed mechanism can increase the number of living-donor transplants by 30%. However, deceased donation has been contributing to greater than 95% of liver donations in the last 15 years in the U.S. Unlike living donation, which can be arranged privately between a patient-donor pair, deceased donor organs are considered national resources (whose allocation is determined by government policy). We focus on deceased donation in this study; the parameters used in our model and their policy implications are likely to remain unaffected with recent promising developments in living donation.

## Model formulation

Consistent with UNOS’ stated principles, our approach is to design an organ distribution policy that equalizes s/d ratios across transplant centers, and thus, mitigates geographical disparities. We start by aggregating the historical supply and demand of organs by geographical location for the period of study. We assume that the distribution of organ quality (Appendix A compares transplant organ quality on a four-year dataset used in our study; we find that there are no significant differences in the distribution of organ quality at recovery across the different regions) and the patient’s health characteristics are similar across donor hospitals and transplant centers. While there are certainly differences currently in the patient health characteristics from state to state (e.g., at present, California has a higher proportion of high-MELD candidates than Tennessee), this is largely a function of accumulated disparity over the years; in steady state, the distribution of MELD scores should be similar.

We formulate an Integer Programming model (IP) that uses a neighborhood framework. Each supply location (e.g., a DSA, zip code, or donor hospital, depending on the context) is assigned a unique set of demand locations (a DSA or transplant center), which is referred to as its neighborhood. In a setting where the geographical units of supply and demand are DSAs, a neighborhood of a DSA consists of other DSAs (including itself) with which it shares its organs.

**Fig. 4:**
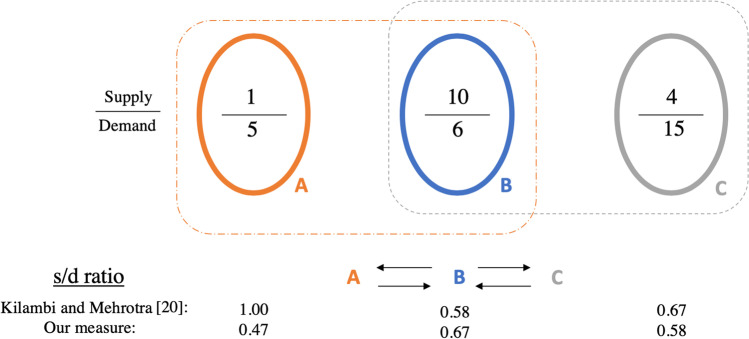
Comparing our s/d ratio measure with that of Kilambi and Mehrotra [20]. Their measure artificially inflates the s/d ratio

Kilambi and Mehrotra [20] pioneered the idea of defining neighborhoods for DSAs. However, their definition of a supply-to-demand ratio at a DSA is somewhat problematic. They model the s/d ratio of a DSA as the ratio of the total supply to the total demand in the DSA’s neighborhood. In other words, they treat all DSAs in that neighborhood as a single unit. However, a DSA can also be part of another neighborhood, which results in the artificial inflation of the s/d ratio. To illustrate, consider three DSAs A (Supply: 1, Demand: 5), B (Supply: 10, Demand: 6), and C (Supply: 4, Demand: 15), as shown in Fig. 4. A shares with B and receives from B; B shares with and receives from both A and C; and C shares with B and receives from B. The neighborhood of A consists of A and B; the neighborhood of B consists of A, B, and C; and the neighborhood of C consists of B and C. Kilambi and Mehrotra compute the s/d ratios of A, B, and C as 1.00 (11/11), 0.58 (15/26), and 0.67 (14/21), respectively. However, in aggregate, the s/d ratio for this three-region system is only 0.58! Further, their objective function is to minimize the absolute deviation of the s/d ratios from a target value (the national average), which effectively treats deviations below the average identically to deviations above the average. Unfortunately, locations with deviations below the average (i.e., lower s/d ratios and higher MMaT scores) have poorer outcomes (greater chances of dying while waiting for a transplant) than locations with deviations above the average. Thus, in a setting where the desire is to minimize disparities, it does not seem appropriate to treat these two deviations identically. By maximizing the worst s/d ratio, our primary focus is on minimizing the deviation below the national average. Finally, we note that our model does not require symmetric organ sharing (which they enforce), giving more flexibility in optimization.

### Supply-demand ratio calculation

First, we define our s/d ratio measure. Recall that we assumed the MELD scores of candidates across geographies are independent and identically distributed (i.i.d.); and when an organ is recovered, all locations in the neighborhood are treated alike. For a given demand location *j* in the neighborhood of supply location *i*, we model the expected supply received (by *j*) from *i* to be proportional to $j^{\prime }$s demand over the total demand competing for $i^{\prime }$s supply. Using this expected allocation of the supply in the example in Fig. 4, we find that 5/11 units of the supply from A are allotted to A, and 6/11 units of the supply from A are allotted to B. Similarly, (5/26)×10, (6/26)×10, and (15/26)×10 units of the supply from B are allotted to A, B, and C, respectively. Finally, (6/21)×4 units of the supply from C are allotted to B, and (15/21)×4 units of the supply from C are allotted to C. Dividing the expected supply provided to each location by its demand, we find the s/d ratios of 0.47, 0.67, and 0.58 for A, B, and C, respectively, with our measure. Using the notation described in Table 2, we formally calculate,

$$\begin{array}{@{}rcl@{}} \text{Expected supply from }i \text{ to } j = \frac{d_{j}}{{\sum}_{k=1}^{N_{dem}} d_{k} x_{ik}} s_{i} x_{ij} \end{array}$$

**Table 2:** Model notation

Notation	Description
$i\in \mathcal {I} =\{1,...,N_{sup}\}$	Supply locations (e.g., a DSA, zip code, or donor hospital)
$j\in \mathcal {J} =\{1,...,N_{dem}\}$	Demand locations (e.g., a DSA or transplant center)
**Parameters**:	
*s*_*i*_	Number of livers from deceased donors recovered (or supply) at *i*
*d*_*j*_	Number of incident waiting list additions (or demand) at *j*
*τ*_*ij*_	Distance between locations *i* and *j*
*τ*_*max*_	Maximum permissible distance from a supply location to a demand location
*c*_*j*_	Number of transplant centers in demand location *j*
$c_{i}^{(r)}$	Number of transplant centers that are ≤ *r* distance units away from supply location *i*
*c*_*min*_	Minimum number of transplant centers with which a supply location must share its organs
$\lambda _{[S-1]}^{*}$	Minimum s/d ratio value to be used in Stage 2 optimization
$s_{ij}^{(r)}$	Apportioned share of organs from *i* to *j* when the farthest demand location in *i*^′^*s* neighborhood is *r* units away
**Decision variables**:	
*x*_*ij*_ (General model)	1 if *i* shares its organs with *j*, and 0 otherwise
*x*_*ir*_ (Set-partitioning model)	1 if the farthest member in the neighborhood of *i* is *r* units away from *i*, and 0 otherwise
*λ*	Minimum s/d ratio for an allocation
β	Maximum s/d ratio for an allocation

To determine the overall supply-to-demand ratio, we first sum the expected supply over all supply locations and then divide by $j^{\prime }$s demand, *d*_*j*_ giving:

$$\begin{array}{@{}rcl@{}} \text{s/d ratio at }j = \sum\limits_{i=1}^{N_{sup}} \frac{1}{{\sum}_{k=1}^{N_{dem}} d_{k} x_{ik}} s_{i} x_{ij} \end{array}$$

We note that the way we calculate the expected s/d ratio does not account for organs that a DSA may receive only due to national sharing. However, these organs are generally a very small fraction (less than 4% in a four-year dataset used in our study) and should not significantly impact the s/d ratios realized in practice.

### General model

We now describe our model, which solves the problem in two stages. In Stage 1, we apply the *maximin* equity principle to maximize the performance of the worst demand location (i.e., we maximize the value of the lowest s/d ratio across all demand locations). In Stage 2, we reduce the disparity among the different demand locations. To do this, we minimize the disparity between the best and worst demand locations, while ensuring the s/d ratio of the worst demand location remains at the optimum value obtained from the Stage 1 optimization. We now present the Mixed-Integer Linear Programs (MIPs) for the different stages.

#### Stage 1 formulation

In Stage 1, we seek to maximize the s/d ratio of the worst demand location.

1 $$\begin{array}{@{}rcl@{}} \textbf{[S-1]} && {\text{Maximize }\lambda} \end{array}$$

2 $$\begin{array}{@{}rcl@{}} \text{subject to:} && \qquad\lambda \leq \sum\limits_{i=1}^{N_{sup}} \frac{1}{{\sum}_{k=1}^{N_{dem}} d_k x_{ik}} s_i x_{ij} \quad \forall j\in \mathcal{J} \end{array}$$

3 $$\begin{array}{@{}rcl@{}} & &x_{ij} \tau_{ij} \leq \tau_{max} \qquad\qquad\qquad\forall i\in \mathcal{I},j\in \mathcal{J} \end{array}$$

4 $$\begin{array}{@{}rcl@{}} & &x_{ij} = 1\qquad\qquad\qquad \forall i=j, i\in \mathcal{I}, j\in \mathcal{J} \end{array}$$

5 $$\begin{array}{@{}rcl@{}} && \sum\limits_{j=1}^{N_{dem}} c_j x_{ij} \geq c_{min} \qquad\qquad\qquad\qquad\forall i \in \mathcal{I} \end{array}$$

6 $$\begin{array}{@{}rcl@{}} && {\text{Contiguity constraints}} \end{array}$$

7 $$\begin{array}{@{}rcl@{}} && x_{ij} \in \{0,1\}\qquad\qquad \qquad\quad\forall i\in \mathcal{I}, j\in \mathcal{J} \end{array}$$

**Fig. 5:**
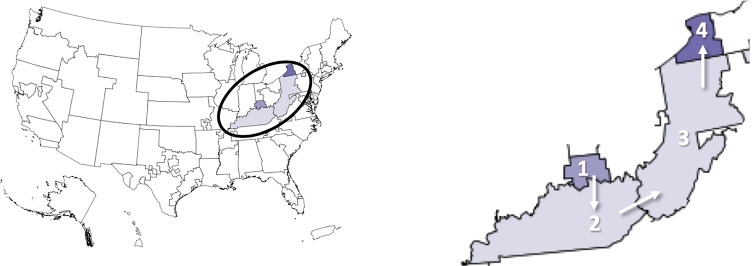
Illustration of sharing and receiving contiguity. If *x*_14_ = 1, with sharing contiguity, *x*_12_ = *x*_13_ = 1; and with receiving contiguity *x*_24_ = *x*_34_ = 1

Constraint (2) models *λ* as the lower bound of the s/d ratios across all the demand locations, and the objective is to maximize this lower bound. Constraint (3) limits the size of the neighborhood (by limiting how far an organ can be transported for transplantation); constraint (4) implies that if a supply-and-demand location coincide (e.g., a DSA or zip code that has both a donor hospital and a transplant center), it must share with itself; and constraint (5) ensures that there are at least *c*_*min*_ transplant centers in a neighborhood.^8^ We also include contiguity constraints to ensure that the designed neighborhoods are contiguous and somewhat compact in shape. This is enforced by an adjacency matrix, which describes locations that are geographically adjacent to each other, and two types of contiguity constraints. Sharing contiguity ensures that if location *r* supplies organs to location *t* (which is not adjacent to it), then all locations between *r* and *t* also receive organs from location *r*. Receiving contiguity ensures that if location *r* supplies organs to location *t* (which is not adjacent to it), then all locations between *r* and *t* also supply organs to location *t*. Figure 5 illustrates receiving and sharing contiguity, ensuring that if location 1 shares its organs with location 4, locations 2 and 3 also share their organs with location 4, and locations 2 and 3 also receive organs from location 1. Appendix B describes flow-based mathematical constraints, applying Shirabe’s [28] approach, which can be used to enforce sharing and receiving contiguity with any geographical shapes, as well as a linearization of constraint (2) in the nonlinear integer programming model [S-1].

#### Stage 2 formulation

In Stage 2, we minimize the maximum absolute difference of the s/d ratios among demand locations. This is achieved by constraining the lowest s/d ratio value to be greater than or equal to the Stage 1 objective ${\lambda _{[S-1]}^{*}}$, and by minimizing the maximum s/d ratio value across all demand locations.

8 $$\begin{array}{@{}lcl@{}} \textbf{[S-2]} && {\text{Minimize }\quad\upbeta} \\ \text{subject to:} && \upbeta \geq \sum\limits_{i=1}^{N_{sup}} \frac{1}{{\sum}_{k=1}^{N_{dem}} d_k x_{ik}} s_i x_{ij} \qquad \forall j\in \mathcal{J} \end{array}$$

9 $$\begin{array}{@{}rcl@{}} && \lambda \geq \lambda_{[S-1]}^* \end{array}$$

10 $$\begin{array}{@{}rcl@{}} && \text{All constraints from [S-1]} \end{array}$$

The optimal values of *x*_*ij*_ obtained by optimizing [S-1], followed by [S-2], are used to construct the new optimized geographical scheme.

### Circular contiguity and a set-partitioning model

**Fig. 6:**
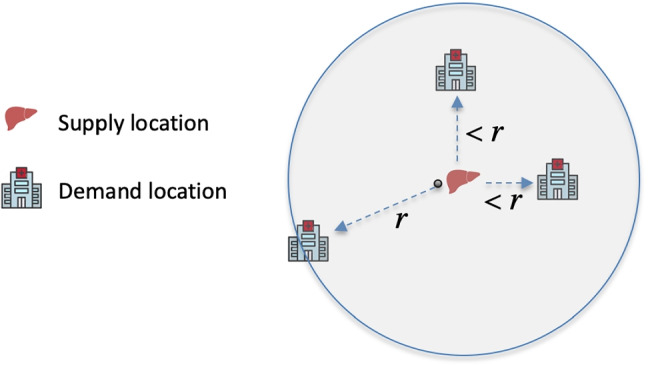
Illustration of circular contiguity: If a neighborhood is *r* units in radius around the supply location, then all demand locations within *r* units must be in the neighborhood

One of the chief complaints in the liver and lung lawsuits was that a candidate receiving the transplant organ may be geographically farther away from the donated organ than another sicker candidate. In other words, neighborhood boundaries that allow an organ to be transported farther away to a less sick candidate than a closer sicker candidate (because the sicker candidate is outside the neighborhood) goes against generally accepted perceptions of fairness. This notion suggests that we consider (roughly) *circular contiguity* for neighborhoods. If the radius of a neighborhood is *r* units around the supply location, then all demand locations within *r* units away are in the neighborhood (Fig. 6).

Circular contiguity allows for a more computationally tractable reformulation of the previous model. For a neighborhood of a given radius *r*, one can easily calculate (a priori) the amount of supply allocated to each demand location in the neighborhood. This enables us to reformulate [S-1] and [S-2] linearly as *Set-Partitioning Problems*, which also makes them scalable. In the set-partitioning formulation, *x*_*ir*_ is a binary decision variable that takes a value 1 if the radius of the neighborhood of *i* is *r* units (all demand locations ≤ *r* units from *i* are part of the neighborhood), and 0 otherwise.

#### Stage 1 formulation

11 $$\begin{array}{@{}rcl@{}} \textbf{[SP-1]} && {\text{Maximize }\quad\lambda} \end{array}$$

12 $$\begin{array}{@{}rcl@{}} \text{subject to:} && \lambda \leq \sum\limits_{i=1}^{N_{sup}} \sum\limits_{r \in \mathcal{R}_i} \frac{x_{ir} s_{ij}^{(r)}}{d_j} \qquad \forall j\in \mathcal{J} \end{array}$$

13 $$\begin{array}{@{}rcl@{}} && \sum\limits_{r \in \mathcal{R}_i} x_{ir} = 1\quad\quad\quad\,\,\, \forall i\in \mathcal{I} \end{array}$$

14 $$\begin{array}{@{}rcl@{}} && \sum\limits_{r \in \mathcal{R}_i} c_i^{(r)} x_{ir} \geq c_{min}\quad \forall i\in \mathcal{I} \end{array}$$

15 $$\begin{array}{@{}rcl@{}} && x_{ir} \in \{0,1\} \forall i\in \mathcal{I},\quad\, \forall r\in \mathcal{R}_i \end{array}$$

For a given radius *r*, $s_{ij}^{(r)}$ denotes the apportioned share of *i*^′^*s* organs that are expected to be offered to location *j*. In other words, $s_{ij}^{(r)}= \frac {d_{j}}{{\sum }_{k:\tau _{ik}\leq r} d_{k}}s_{i}$, which can be precomputed for a given radius *r*. Note that for a given supply location *i*, we do not need to consider a continuum of possible neighborhood radii. Rather (because this apportionment of organs will only change when a new demand location is added to the neighborhood), we only need to consider a finite set of values of *r* that correspond to the distance from *i* to each of the other demand locations that are within *τ*_*max*_. In [SP-1], the set $\mathcal {R}_{i}$ contains the possible values of *r* created accordingly. Constraint (12) models *λ* as the lower bound of the s/d ratios across all demand locations; and the objective is to maximize this lower bound. Constraint (13) allows one assignment of *r* to each supply location; and constraint (14) ensures a minimum number of transplant centers in the neighborhood.

#### Stage 2 Formulation

Once the optimal solution $\lambda _{[SP-1]}^{*}$ to [SP-1] is obtained, we can solve [SP-2] to minimize the maximum s/d ratio while ensuring that the minimum s/d ratio remains at least $\lambda _{[SP-1]}^{*}$.

16 $$\begin{array}{@{}lcl@{}} \textbf{[SP-2]} && {\text{Minimize }\quad\upbeta} \\ \text{subject to:} && \upbeta \geq \sum\limits_{i=1}^{N_{sup}} \sum\limits_{r \in \mathcal{R}_i} \frac{x_{ir} s_{ij}^{(r)}}{d_j} \qquad \forall j\in \mathcal{J} \end{array}$$

17 $$\begin{array}{@{}lcl@{}} && \lambda \geq \lambda_{[SP-1]}^{*} \end{array}$$

18 $$\begin{array}{@{}rcl@{}} &&\text{All constraints from [SP-1]} \end{array}$$

## Data and results

This study used data from SRTR. The SRTR data system includes data on all donors, waitlisted candidates, and transplant recipients in the U.S., submitted by members of the OPTN. The Health Resources and Services Administration (HRSA), U.S. Department of Health and Human Services, provides oversight to the activities of OPTN and SRTR contractors.

In the data, encompassing the four years starting from July 2013 and ending in June 2017, the supply or the total number of livers (from deceased donors) donated from all donor hospitals in the U.S. is 26,899. The patient pool is dynamic: new patients enlist, waiting candidates die or become too sick for transplant and are removed, and the MELD scores get updated periodically. We measure demand (44,959) as the total incident^9^ adult patients whose MELD scores became at least 15 during the four years, which gives a national s/d ratio of 0.5983. There are two reasons for excluding low-MELD patients from the demand: (1) patients with MELD scores < 15 have no survival benefit from transplantation [24]; therefore, our demand measure is less sensitive to the number of low-MELD patients added to the waiting list and (2) transplant centers differ in their practices of listing low-MELD patients, across the country (which would create an artificial increase in demand for a transplant center listing low-MELD patients compared to a transplant center that does not). In practice, the fraction of transplants to low-MELD patients is relatively very low—about 1.08% (in the four years encompassing our study), supporting the decision to exclude them.

We apply the set-partitioning optimization model to two versions of the data: a zip-code cluster version where the supply locations are zip-code clusters (clustered by the first three digits and first four digits) and the demand locations are the 142 transplant centers, and a DSA version where the supply and demand locations are the DSAs. We restrict *r* (radius around the supply locations) within the range 150 NM to *τ*_*max*_ for every $\mathcal {R}_{i}$, constraining the minimum and maximum size of the neighborhoods. We set *c*_*min*_ = 3, ensuring that at least three transplant centers are present in a neighborhood.^10^ We used R 3.5.1 and the commercial solver Gurobi 8.1.1 to solve the set-partitioning optimization models on a 3.2 GHz 6-Core Intel Core i7 iMac with 32 GB RAM.

### Zip-code cluster version

The locations of the zip codes and transplant centers are indicated by their latitude and longitude values. To calculate the distance between a three-digit (four-digit) zip-code cluster and a transplant center, we first find the centroid of the zip-codes in the cluster having the same first three digits (four digits) and then use the “geosphere” package in R to calculate the shortest distance between two points (centroid of the zip cluster and transplant center) according to the “Vincenty (ellipsoid)” method.

There are a total of 641 three-digit and 1,380 four-digit zip-code clusters with the supply in our data.^11^ We vary *τ*_*max*_ from 350 NM to 700 NM in steps of 50 NM. We do not include the zip codes in Hawaii and Puerto Rico in our analysis, given that they are more than 1,000 miles from the transplant centers in the mainland U.S. Consistent with the current policy zip codes in Alaska are considered to be situated at the Seattle Tacoma Airport in Washington State. We require that the minimum radius of a neighborhood be 150 NM (to try and keep parity with the radius of the innermost concentric circle in the Acuity Circle policy). Because a transplant hospital may not necessarily be exactly 150 NM from a zip-code cluster, this is enforced by ensuring that the closest transplant center greater than or equal to 150 NM away is included in the neighborhood, unless it is greater than *τ*_*max*_ miles away. Appendix C provides computational details—the problem size, running times, cutting planes, simplex iterations, etc.—for the set-partitioning model on the four-digit zip-clusters.

Table 3 provides a comparison of the s/d ratios. To compare against the fixed radius type of policy currently in place (i.e., Acuity Circle), we also computed the s/d ratio for homogeneous radii circles by fixing the radius of each zip-code cluster to *τ*_*max*_. Compared to the heterogeneous radius circle policy, the “one-size-fits-all” fixed radius policy does a poor job at equalizing the s/d ratios across transplant centers. The heterogeneous circle policy at *τ*_*max*_ = 500 NM is able to keep the ratio at transplant centers between 0.55 and 0.60 (compared to the national s/d ratio of 0.5983), while the fixed 500 NM radius circle policy has an s/d ratio variation between 0.37 and 0.84.

**Table 3:** Comparison of the s/d ratios between fixed and heterogeneous circles (supply and demand locations are zip-code clusters and transplant centers (TCs), respectively)

Allocation policy	s/d ratio		Maximum (Median) s/d ratio
	Range	Std. deviation	difference within 150 NM from TC
*τ*_*max*_ = 350 NM			
Fixed radius circles (Three-digit zip)	0.39-1.09	0.123	0.59920 (0.04611)
Fixed radius circles (Four-digit zip)	0.38-1.09	0.123	0.60235 (0.04559)
Three-digit zip-code cluster model	0.51-0.88	0.098	0.33043 (0.05047)
Four-digit zip-code cluster model	0.51-0.88	0.103	0.33813 (0.07085)
*τ*_*max*_ = 400 NM			
Fixed radius circles (Three-digit zip)	0.37-0.85	0.112	0.23255 (0.03690)
Fixed radius circles (Four-digit zip)	0.37-0.84	0.112	0.22818 (0.03633)
Three-digit zip-code cluster model	0.53-0.62	0.033	0.08571 (0.00042)
Four-digit zip-code cluster model	0.53-0.61	0.030	0.07763 (0.00028)
*τ*_*max*_ = 450 NM			
NM Fixed radius circles (Three-digit zip)	0.38-0.88	0.124	0.20629 (0.02900)
Fixed radius circles (Four-digit zip)	0.38-0.87	0.124	0.19770 (0.02048)
Three-digit zip-code cluster model	0.54-0.61	0.023	0.05277 (0.00108)
Four-digit zip-code cluster model	0.54-0.61	0.024	0.06125 (0.00043)
*τ*_*max*_ = 500 NM			
Fixed radius circles (Three-digit zip)	0.37-0.84	0.137	0.20941 (0.03632)
Fixed radius circles (Four-digit zip)	0.37-0.84	0.137	0.20851 (0.04489)
Three-digit zip-code cluster model	0.55-0.60	0.022	0.04621 (0.00009)
Four-digit zip-code cluster model	0.55-0.60	0.021	0.04922 (0.00025)
*τ*_*max*_ = 550 NM			
Fixed radius circles (Three-digit zip)	0.37-0.91	0.145	0.17331 (0.03808)
Fixed radius circles (Four-digit zip)	0.36-0.91	0.146	0.17213 (0.03882)
Three-digit zip-code cluster model	0.55-0.60	0.020	0.05070 (0.00029)
Four-digit zip-code cluster model	0.55-0.60	0.019	0.04387 (0.00025)
*τ*_*max*_ = 600 NM			
Fixed radius circles (Three-digit zip)	0.34-0.97	0.152	0.17767 (0.04866)
Fixed radius circles (Four-digit zip)	0.34-0.96	0.152	0.17819 (0.04473)
Three-digit zip-code cluster model	0.55-0.60	0.018	0.05407 (0.00113)
Four-digit zip-code cluster model	0.55-0.60	0.018	0.03613 (0.00015)
*τ*_*max*_ = 650 NM			
Fixed radius circles (Three-digit zip)	0.33-0.94	0.152	0.16743 (0.02449)
Fixed radius circles (Four-digit zip)	0.33-0.93	0.152	0.17091 (0.02457)
Three-digit zip-code cluster model	0.55-0.60	0.017	0.05049 (0.00016)
Four-digit zip-code cluster model	0.55-0.60	0.018	0.03336 (0.00012)
*τ*_*max*_ = 700 NM			
Fixed radius circles (Three-digit zip)	0.32-0.94	0.145	0.17881 (0.04773)
Fixed radius circles (Four-digit zip)	0.32-0.94	0.145	0.18275 (0.04654)
Three-digit zip-code cluster model	0.55-0.60	0.016	0.05100 (0.00010)
Four-digit zip-code cluster model	0.55-0.60	0.017	0.03402 (0.00007)

We also examine the difference in the s/d ratio of nearby transplant centers (defined as being within 150 NM). Table 3 provides both the maximum and median values of this difference. As is evident, in the heterogeneous circles policy, the value of the s/d ratio at nearby transplant centers is very similar—which can hopefully lead to more equitable transplant outcomes in nearby transplant centers. For most of the transplant centers, the difference in the s/d ratio is at the scale of 10^− 4^, as indicated by the median values.

**Fig. 7:**
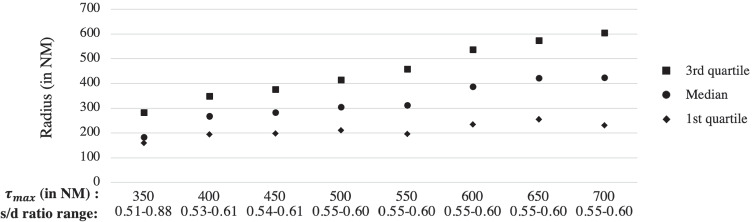
Quartiles of radii in the four-digit zip-code cluster models

As we increase *τ*_*max*_ from 350 NM to 700 NM, the minimum s/d ratio increases, and the range of the s/d ratio decreases. When *τ*_*max*_ = 400 NM, the s/d ratio range is already quite narrow at 0.53-0.61, and once *τ*_*max*_ = 500 NM, the s/d ratio range stays steady at 0.55-0.60. Figure 7 shows the quartiles of the radii when using four-digit zip-code clusters. When *τ*_*max*_ is 500 NM, the first, second, and third quartiles of the radii are 211, 305, and 415 NM, respectively. Compared to the fixed radius circle policy, the heterogeneous radii circle policy achieves an equalization in the s/d ratio (near the national average) while keeping transport distances lower. This has an added benefit. Because the radii of the circles are smaller, each donor zip-code cluster on average has 24 (median of 20) transplant centers, as compared to the fixed radii circles that have on average 39 transplant centers (median of 43). The logistics of a donor hospital (zip-code cluster) coordinating with a smaller number of transplant centers can be much simpler. One may wonder whether fixed population circles (i.e., the radius of the circle around each transplant center is set so that they all cover the same number of people) would reduce disparity. Using the s/d metric defined and introduced in this paper, Haugen et al. [17] analyze the disparity in the s/d ratios across transplant centers with fixed population circles. They find that circles covering a population of 12 million individuals provides s/d values ranging from 0.27 to 2.14. Increasing the size of the circles to cover 50 million individuals decreases the s/d variation to 0.43–1.01.

To check whether our solution is robust to variations in the supply and demand across time, using the optimal radii obtained with the four years of data, we recalculate the s/d ratio range, skipping one year of (supply and demand) data at a time. We find that, on average, the minimum (absolute) s/d ratio changes by 0.016 points, and the maximum (absolute) changes by 0.018 points (based on *τ*_*max*_ = 500, 550, 600, 650, and 700 NM), which indicates that the results are fairly robust to variations in the data.

Given that the current implementation of LSAM does not support schemes based on zip-code clusters, we could not evaluate our zip-code based allocation policy via the LSAM simulation model. Instead, we use the results of the DSA version described in the next section and run the LSAM simulation on the neighborhoods it generates to evaluate the effectiveness of our allocation policy in reducing geographical disparity.

### DSA version

Using DSAs as the geographical unit preserves the existing important relationships between donor hospitals and the OPO in each DSA. If indeed, the court rules in a manner that reinstates DSAs as a geographical unit, then our method shows how they could share organs to achieve equitable outcomes with regard to the s/d ratio.

The distance between any two DSAs *i* and *j*, *τ*_*ij*_, is calculated as the mean of the transplant-volume-weighted distance between donor hospitals in DSA *i* and the transplant centers in DSA *j*, and the reverse. Because six DSAs do not have a transplant hospital, there are 58 DSAs with supply and 52 DSAs with demand. Consistent with Gentry et al. [13] and Kilambi and Mehrotra [20], we allow (as exceptions to *τ*_*max*_) the DSAs located in Hawaii and Puerto Rico to share and receive organs from other DSAs located in California and Oregon, and Florida, respectively.

Table 4 summarizes the results for *τ*_*max*_ set to 500 NM, 600 NM, and 700 NM, and compares it with the prior 11-region system and other proposed geographical allocation policies. As is evident, our model produces a neighborhood that results in the narrowest range of s/d ratios across DSAs: 0.15 when *τ*_*max*_ = 500 NM, 0.13 when *τ*_*max*_ = 600 NM, and 0.10 when *τ*_*max*_ = 700 NM, as compared to 0.34 (OPTN 11 regions), 0.17 [13, 8 districts], and 0.64 [20]. Our model also produces relatively more uniform and smaller-sized neighborhoods. It does not contain any unusually large neighborhoods (as evidenced by the value of *τ*_*max*_). Our solutions have a fair degree of reciprocity (that is, if DSA *i* shares its organs with DSA *j*, then DSA *j* shares its organs with DSA *i*). About 56.0% of DSA pairs had reciprocity when *τ*_*max*_ = 500 NM, 62.1% when *τ*_*max*_ = 600 NM, and 52.7% when *τ*_*max*_ = 700 NM. Further, the average distance of the farthest DSAs in the neighborhoods ($\bar {\tau }$) is much smaller than that of Gentry et al. [13] and Kilambi and Mehrotra [20], and is comparable with OPTN 11 regions. The maximum s/d ratio difference among adjacent DSAs is also reduced significantly. For example, with *τ*_*max*_ = 700 NM, the maximum difference of the s/d ratio among adjacent DSAs is 0.096, much smaller compared to OPTN 11 regions (0.228).

**Table 4:** Comparison of the s/d ratios among different allocation policies in the DSA version (supply and demand locations are 58 DSAs and 52 DSAs, respectively). *τ*_*max*_ and $\bar {\tau }$ represent the maximum and average distance, respectively, of the farthest DSA in a neighborhood/region/district in each allocation policy

Allocation policy	s/d ratio		${\tau _{max}}, \bar {\tau }$	Max. (Median) s/d ratio
	Range	Std. deviation	(in NM)	difference among adjacent DSAs
OPTN 11 regions	0.42-0.76	0.109	843, 401	0.228 (0.117)
Gentry et al. [13]	0.52-0.69	0.054	975, 569	0.120 (0.036)
Kilambi and Mehrotra [20]	0.35-0.99	0.157	1380, 666	0.615 (0.246)
[SP-2], *τ*_*max*_ = 500 NM	0.50-0.65	0.054	500, 349	0.151 (0.086)
[SP-2], *τ*_*max*_ = 600 NM	0.52-0.65	0.051	600, 409	0.132 (0.077)
[SP-2], *τ*_*max*_ = 700 NM	0.53-0.63	0.033	700, 422	0.096 (0.036)

**Fig. 8:**
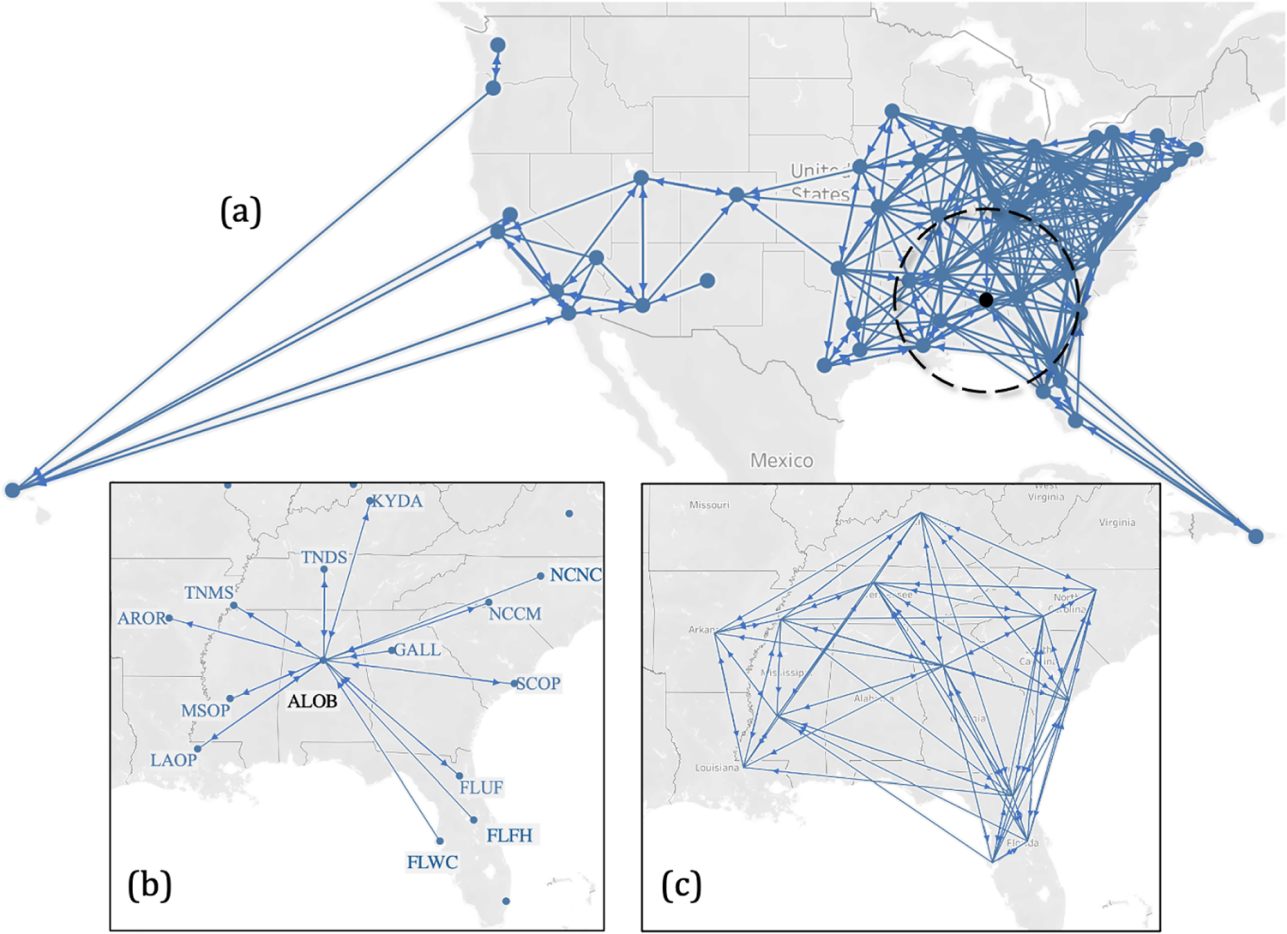
(**a**) Illustration of optimized neighborhoods when *τ*_*max*_ = 500. (**b**) DSAs to which ALOB shares and receives. (**c**) Sharing and receiving between DSAs identified in (**b**)

Table 5 presents the s/d ratios for each DSA in the different proposals. This allows a deeper examination of how each DSA is affected by the proposed reallocations. The maximum and minimum s/d ratio values in every proposal are highlighted in bold. Appendix D describes the DSA neighborhoods obtained by our models for *τ*_*max*_ = 500, 600, and 700 NM, respectively. Figure 8a depicts the neighborhoods (when *τ*_*max*_ = 500) using a directed graph.^12^ An arc from a node (i.e., DSA) *i* to a node *j* means that DSA *i* is sharing its organs with DSA *j*. In the event of reciprocity between DSA’s *i* and *j*, the link between the two nodes is bidirectional. It is interesting to observe that in the mainland U.S., the DSA CORS (which comprises Colorado and Wyoming) forms a cut node (i.e., its removal separates the graph representing the neighborhood into two components). Although there are additional arcs (and sharing between DSAs) with *τ*_*max*_ = 600 and 700 NM, CORS remains a cut node separating the DSAs to the east and west. This suggests that sharing between DSAs largely occurs exclusively between DSAs to the east of CORS, and exclusively between DSAs to the west of CORS (i.e., DSAs to the east of CORS do not share with DSAs to the west of CORS and vice versa). Given that there is a lot of information packed into Fig. 8a, Fig. 8b focuses on the neighborhood of DSA ALOB. It shows the DSAs with which ALOB shares its organs and also shows which DSAs share their organs with ALOB. Figure 8c provides a few additional details and adds in sharing and receiving between the DSAs identified in Fig. 8b (it excludes information about sharing and receiving between the 14 DSAs in the figure and the remaining DSAs).

**Table 5:** Comparison of the s/d ratios among different DSA-based allocation policies (supply and demand locations are 58 DSAs and 52 DSAs, respectively)

DSA	s/d ratio							
	Local, or	OPTN 11 regions	Gentry et al. [13]	Kilambi and Mehrotra [20]	[SP-2]			
	no sharing	*τ*_*max*_ = 843 NM	*τ*_*max*_ = 975 NM	*τ*_*max*_ = 1380 NM	*τ*_*max*_:	500 NM	600 NM	700 NM
ALOB	0.72	**0.76**	0.61	0.56		0.62	**0.65**	**0.63**
AROR	0.97	**0.76**	0.61	0.96		**0.65**	**0.52**	0.58
AZOB	0.55	0.52	0.54	0.88		0.53	0.53	0.59
CADN	0.38	0.52	**0.52**	0.45		0.51	**0.52**	**0.53**
CAOP	0.39	0.52	**0.52**	0.5		0.54	0.53	**0.53**
CASD	0.55	0.52	**0.52**	**0.35**		**0.5**	0.53	0.59
CORS	0.37	0.64	0.54	0.77		0.51	0.54	**0.53**
CTOP	0.95	**0.42**	0.57	0.4		0.56	0.62	0.59
DCTC	0.58	0.57	0.57	0.46		0.64	0.64	**0.63**
FLFH	1.3	**0.76**	0.61	0.65		0.54	**0.52**	0.62
FLMP	0.5	**0.76**	0.61	0.65		0.61	**0.65**	0.61
FLUF	0.47	**0.76**	0.61	0.81		**0.65**	0.58	0.61
FLWC	**1.98**	**0.76**	0.61	0.65		0.64	**0.52**	0.62
GALL	0.72	**0.76**	0.57	**0.99**		**0.65**	**0.65**	0.62
HIOP	0.97	0.66	**0.52**	0.37		0.63	0.64	0.54
IAOP	1.23	0.64	0.64	0.58		0.62	0.64	0.61
ILIP	0.69	0.55	**0.69**	0.62		**0.65**	0.62	0.62
INOP	0.78	0.66	**0.69**	0.67		0.62	0.63	0.59
KYDA	0.66	**0.76**	**0.69**	0.69		**0.65**	0.64	0.6
LAOP	0.55	**0.76**	0.61	0.64		0.63	**0.65**	**0.63**
MAOB	0.39	**0.42**	0.57	0.4		0.54	0.61	0.56
MDPC	0.34	0.57	0.57	0.67		0.64	**0.65**	**0.63**
MIOP	0.68	0.66	**0.69**	0.49		0.54	0.64	**0.63**
MNOP	0.4	0.55	0.64	0.53		0.51	0.56	0.56
MOMA	0.71	0.64	0.61	0.73		**0.65**	0.63	**0.63**
MSOP	1.49	**0.76**	0.61	0.56		0.58	0.55	**0.63**
MWOB	1.04	0.64	0.64	0.7		**0.5**	**0.52**	0.56
NCCM	0.73	**0.76**	0.57	0.44		**0.65**	0.64	0.62
NCNC	0.77	**0.76**	0.57	0.63		**0.65**	0.6	0.62
NEOR	0.41	0.64	0.64	0.44		0.51	0.54	**0.63**
NJTO	1.19	0.57	0.57	0.47		**0.65**	**0.65**	**0.63**
NYFL	0.56	**0.42**	**0.69**	0.59		0.53	0.53	0.62
NYRT	**0.31**	**0.42**	0.57	0.47		**0.65**	**0.65**	**0.63**
OHLB	0.47	0.66	**0.69**	0.67		**0.65**	**0.65**	0.62
OHLP	0.9	0.66	**0.69**	0.83		**0.65**	0.61	0.62
OHOV	0.33	0.66	**0.69**	0.51		**0.65**	**0.65**	0.61
OKOP	0.91	0.53	0.64	0.81		0.58	**0.52**	**0.63**
ORUO	0.71	0.66	**0.52**	0.62		0.62	**0.65**	0.58
PADV	0.62	0.57	0.57	0.6		0.62	**0.65**	**0.63**
PATF	0.58	0.57	**0.69**	0.83		0.64	0.59	**0.63**
PRLL	1.69	**0.76**	0.57	0.56		0.54	0.6	**0.53**
SCOP	1.02	**0.76**	0.57	0.38		**0.65**	0.62	0.62
TNDS	1.17	**0.76**	**0.69**	0.77		**0.65**	0.64	**0.63**
TNMS	0.36	**0.76**	0.61	0.85		**0.65**	0.64	0.62
TXGC	0.36	0.53	0.61	0.52		0.64	0.58	**0.63**
TXSA	0.5	0.53	0.61	0.44		0.53	**0.52**	**0.53**
TXSB	0.77	0.53	0.61	0.5		0.64	0.55	0.62
UTOP	0.53	0.52	0.54	0.47		0.54	0.55	0.56
VATB	0.6	**0.76**	0.57	0.85		0.63	**0.65**	0.62
WALC	0.6	0.66	**0.52**	0.6		0.62	0.62	0.55
WIDN	0.4	0.55	**0.69**	0.5		**0.5**	0.54	0.62
WIUW	0.61	0.55	**0.69**	0.72		0.62	0.63	**0.63**

The computational benefit of [SP-1] over [S-1] is easily seen in the DSA version. For example, when *τ*_*max*_ = 500 NM, the size of [S-1] using only sharing contiguity was 16,286 rows and 18,883 columns, and the MIP gap (MIP gap = $\frac {|Objective bound - Objective value|}{|Objective value|}$) was 1.19% after two hours of running time. Meanwhile, the size of [SP-1] was 110 rows and 742 columns, and took only 0.66 seconds to reach optimality.

#### Liver simulated allocation model (LSAM) results

Next, we wanted to see how the proposed (DSA-based) allocation policies perform on metrics that policymakers have traditionally examined to evaluate policies, such as the variance of MMaT across geographies, distance traveled, and number of deaths. To this end, we use LSAM to simulate our neighborhoods [SP-2], OPTN 11 regions, Gentry et al. [13] (8 districts), and Kilambi and Mehrotra’s [20] neighborhoods. There are two main inputs to LSAM: (1) patient and organ arrival processes, and (2) the allocation policy that includes geographical schemes and offer prioritization rules.

LSAM uses the historical data of donors and patients to simulate the waitlisted patient’s health state transitions, organ acceptance behavior, and post-transplant survival outcomes. When an organ becomes available, candidates on the waiting list are prioritized for the organ offer as per the allocation policy. When a candidate receives a transplant, the simulation determines the survival time of the transplanted organ and uses this information to determine when in the future the candidate may die or relist. Using LSAM in its current form does have some limitations. It uses a probability acceptance function built on past data, where distance is more strongly correlated with acceptance of an organ due to the lack of broader sharing. It also does not account for organ availability in determining organ acceptance. These limitations may underestimate the effects of broader sharing and the equalization of the s/d ratios. Despite these limitations, it is instructive to use LSAM as a first step in evaluating the potential benefit of the heterogeneous radii circle policy.

In the simulation study (to model broader sharing within a circle), we allow for full sharing of organs to Status 1A/1B and MELD ≥ 15 candidates in the neighborhood or region/district in which the organ is recovered in the first level of allocation. In the next allocation level, the organ is offered nationally to Status 1A/1B, then nationally to candidates with MELD ≥ 15. Next, it is offered to candidates with MELD < 15 locally (the DSA in which the organ is recovered), then in the neighborhood or region/district, and then nationally before being discarded after 100 offers. The above policy (which we refer to as “Enhanced Share 15”) skips sequences # 2 and 3 of the Share 35 policy described in Table 1. For benchmarking, we also compared using the prior Share 35 policy. We simulated the different DSA-based geographical allocation policies using the organ and patient arrival data, consisting of three years (July 2013 to June 2016). We ran the simulation 10 times (the maximum allowed by LSAM) by resampling the input files.

Table 6 compares the simulation results under Enhanced Share 15. The average number of annual waitlist deaths and total deaths is smallest for [SP-2], *τ*_*max*_ = 700 NM, with a projected savings of 114 lives annually, compared to OPTN 11 regions. The average travel distance, although slightly higher in our allocation policy compared to OPTN 11 regions, is smaller than that of the other policies. To measure the differences between DSAs, we consider the variance of MMaT and the standard deviation of the average organ travel distance across DSAs. The variance of MMaT across the DSAs is smallest in our allocation policies (2.00 when *τ*_*max*_ = 500 NM, 1.98 when *τ*_*max*_ = 600 NM, and 1.61 when *τ*_*max*_ = 700 NM), compared to 7.26, 5.22, and 2.68 in OPTN 11 regions, Gentry et al. [13], and Kilambi and Mehrotra [20], respectively. Because the different proposals vary in their efficiency (travel distance) and fairness (MMaT) metrics, it is instructive to compare the fairness of the proposals with similar efficiency levels. To this end, comparing [SP-2], *τ*_*max*_ = 500 NM against OPTN 11 regions shows a significant reduction in both total deaths and variance of MMaT. Similarly, comparing [SP-2], *τ*_*max*_ = 700 NM against Gentry et al. [13] and Kilambi and Mehrotra [20] shows a significant reduction in the variance of MMaT. Overall, we see that greater fairness can be achieved by DSA-based geographical allocation policies that equalize s/d ratios. The standard deviation of the average travel distance across the DSAs (Hawaii and Puerto Rico are excluded from our distance analysis) in our allocation policies is less than half that of the others. This finding indicates that there is less disparity in the travel distance between DSAs because our neighborhoods have relatively similar sizes.

**Table 6:** Comparison of LSAM simulation results for DSA-based allocation policies under Enhanced Share 15

Allocation policy	Avg. (Quartiles)	Waitlist	Total	Across DSAs	
	travel distance	deaths	deaths	Var/Avg	Std. deviation of avg
	(in NM)	(annual)	(annual)	of MMaT	travel distance (NM)
OPTN 11 regions	258 (75, 194, 347)	1411.6	3658.8	7.26/30.0	109
Gentry et al. [13]	309 (101, 226, 429)	1376.1	3600.0	5.22/31.1	124
Kilambi and Mehrotra [20]	305 (124, 240, 395)	1348.2	3555.4	2.68/31.6	142
[SP-2], *τ*_*max*_ = 500 NM	258 (112, 220, 341)	1356.4	3567.7	2.00/31.3	56
[SP-2], *τ*_*max*_ = 600 NM	283 (125, 251, 384)	1343.6	3551.4	1.98/31.7	55
[SP-2], *τ*_*max*_ = 700 NM	293 (125, 250, 399)	1343.4	3544.6	1.61/31.7	64

Table 7 compares the LSAM simulation results under Share 35. We note that our neighborhoods are optimized under the assumption of full sharing, which is closer to Enhanced Share 15 than Share 35; and thus, the full benefits of the improved MMaT are less likely to be seen. Given that there is less sharing under Share 35 (organ offers are restricted to within-DSA patients (15 ≤ *MELD* < 35) before being offered broadly (neighborhood or region/district and nationally)), the average travel distance significantly decreased, and the number of waitlist and total deaths increased for all policies. Even so, comparing [SP-2], *τ*_*max*_ = 500 NM against OPTN 11 regions shows a significant reduction in both total deaths and variance of MMaT. Similarly, comparing [SP-2], *τ*_*max*_ = 600 NM against Gentry et al. [13] and Kilambi and Mehrotra [20] shows a significant reduction in the variance of MMaT. Similar to Enhanced Share 15, we observe again that the standard deviation of the average travel distance (across DSAs) is much lower for our allocation policies.

**Table 7:** Comparison of LSAM simulation results for DSA-based allocation policies under Share 35

Allocation policy	Avg. (Quartiles)	Waitlist	Total	Across DSAs	
	travel distance	deaths	deaths	Var/Avg	Std. deviation of avg.
	(in NM)	(annual)	(annual)	of MMaT	travel distance (NM)
OPTN 11 regions	199 (20, 105, 258)	1455.5	3744.9	13.66/28.5	88
Gentry et al. [13]	231 (25, 130, 314)	1419.5	3696.4	10.49/29.5	102
Kilambi and Mehrotra [20]	230 (32, 150, 309)	1389.0	3656.3	11.87/30.2	104
[SP-2], *τ*_*max*_ = 500 NM	203 (29, 142, 291)	1399.9	3664.8	10.30/29.7	57
[SP-2], *τ*_*max*_ = 600 NM	221 (32, 157, 326)	1384.8	3645.2	8.80/30.3	57
[SP-2], *τ*_*max*_ = 700 NM	233 (36, 162, 344)	1397.3	3636.4	10.04/30.3	64

Ultimately, comparing our allocation policy *τ*_*max*_ = 500 NM under Enhanced Share 15 against the OPTN 11 regions under Share 35 shows that a drastic reduction in the variance of MMaT across DSAs (from 13.66 to 2.00) and deaths (from 3,745 to 3,568) can be achieved with a modest increase in the average travel distance (from 199 NM to 258 NM).

## Conclusions

We use the Rawlsian *maximin* principle to minimize the variability in deceased donor liver access across geographies. In contrast to the current fixed radius policy, we propose heterogeneous radii circles. The benefit of heterogeneous radii circles is that they account for where the organ supply and demand occur, and adjust the radii of the circles so that each transplant center’s s/d ratio can be close to the national average. Moreover, equalizing the s/d ratios at the transplant centers is achieved without a significant increase in anticipated travel distance. In fact, the median radius is approximately 305 NM. In other words, the optimization model only increases the radii of donor circles when necessary.

By using a DSA as the geographical unit, we demonstrate that low geographical variation in the s/d ratio can be achieved while maintaining DSA boundaries by judiciously creating neighborhoods for each DSA. An LSAM evaluation of our DSA neighborhoods predicts a significant reduction in the number of deaths, overall variation in MMaT, and average travel distance across DSAs.

As noted earlier, there are limitations of our analysis, as LSAM’s organ acceptance function may not accurately reflect the change in the candidate/transplant center behaviors when organ accessibility and availability changes. For instance, candidates at organ-rich locations might behave more selectively in accepting organs than at locations with low s/d ratios. In a related paper, Akshat et al. [1] develop a patient’s dynamic choice model to analyze his/her strategic response to a policy change. They show that the policy framework developed in this paper (i.e., equalizing s/d ratios across the geography) indeed promotes the greatest geographic equity and transplant efficiency in comparison to the current Acuity Circle policy and the prior Share 35 policy.

In terms of logistical implementation of the heterogeneous circle policy, we have a few suggestions. First, we believe the circles should be defined around the donor location rather than the transplant location (note that in a fixed radius policy, there is no difference between the circles defined around donor and transplant locations, but with heterogeneous circles, there is a difference), or else the issues raised in the lawsuits (i.e., organs being offered to a less sicker candidate who is farther away) would not be addressed. Second, we expect small variations in the supply and demand over time. Hence, we suggest that the optimization model be run occasionally to account for demographic changes.

Our approach can be viewed as a combination of the fixed distance from a donor hospital and a mathematical optimized boundaries framework identified by the Geography Committee. There is considerable debate in the transplant community about using continuous scoring (the third distribution framework identified by the Geography Committee). The following two papers [25, 29] provide an overview of the continuous scoring concept. Rather than a one-size-fits-all framework for continuous scoring, which we do not believe will adequately address geographical inequities, we would recommend a mathematically optimized continuous scoring function that accounts for regional differences in the supply and demand. In ongoing work, our group is developing an optimization model (to equalize supply-to-demand ratios across transplant centers) that uses a continuous function to assign points to patients based on their distance to the donor hospital.

Clearly, the optimization concepts applied to mitigate geographical disparities in the liver transplantation setting could also be applied to other organs. We hope this research will spur similar work in other organ transplantation settings, and thus reduce/mitigate the geographical disparities that are inherent to all of these systems!

## Figures and Tables

**Fig. 9:**
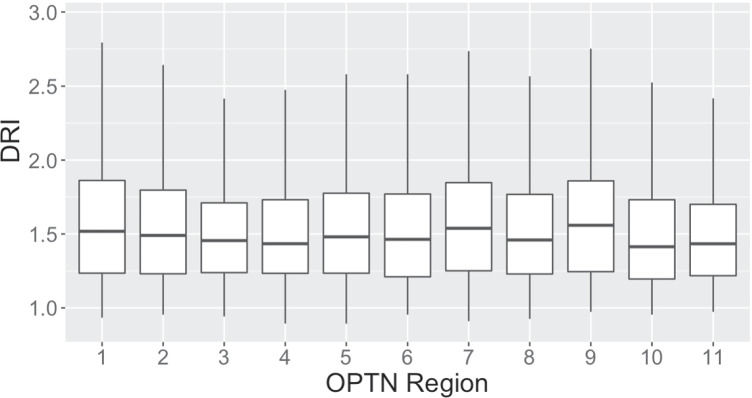
Comparison of organ quality across the regions using the donor risk index (DRI)

## Appendix A: Comparing organ quality

We use the metric, donor risk index (DRI), proposed by Feng et al. [9] to evaluate the quality of the organs in our dataset. This index measures the quality of an organ using demographic factors (age, race, height), cause and type of donor death, sharing type (local/regional/national), and cold ischemia time. A higher DRI is associated with a greater risk of graft failure. Given that we want to assess the quality of the organs at the time of recovery, we exclude cold ischemia time, which depends on the transplant locations and assumes local sharing for an adequate comparison. In Fig. 9, we compare the box plots of DRI across the regions. We see that there are no significant differences in the distributions of organ quality across the regions.

## Appendix B: Linearization of the s/d ratio and contiguity constraints

To linearize the right-hand side of constraint (2) in [S-1], i.e., ${\sum }_{i=1}^{N_{sup}} \frac {1}{{\sum }_{k=1}^{N_{dem}} d_{k} x_{ik}} s_{i} x_{ij}$, we introduce auxiliary variables: *y*_*i**j*_ ≥ 0 and *t*_*i*_ ≥ 0, which are defined as follows.

19 $$\begin{array}{@{}rcl@{}} t_{i} & =& \frac{1}{{\sum}_{k=1}^{N_{dem}}d_{k} x_{ik}} \qquad \forall i\in \mathcal{I} \end{array}$$

20 $$\begin{array}{@{}rcl@{}} y_{ij} & = &t_{i} x_{ij} \qquad \qquad \qquad \forall i\in \mathcal{I}, j\in \mathcal{J} \end{array}$$

Together, they imply:

21 $$\begin{array}{@{}rcl@{}} \lambda \leq \sum\limits_{i=1}^{N_{sup}} s_{i} y_{ij} \qquad \forall j\in \mathcal{J} \end{array}$$

22 $$\begin{array}{@{}rcl@{}} \quad \sum\limits_{k=1}^{N_{dem}} d_{k} y_{ik} = 1 \qquad \forall i \in \mathcal{I} \end{array}$$

A set of linear constraints (22) model equation (19). We note that *t*_*i*_ ≤ 1 and, *y*_*i**j*_ = { 0 if *x*_*i**j*_ = 0*t*_*i*_ if *x*_*i**j*_ = 1. Because *y*_*i**j*_ is a product of two variables and therefore non-linear, the following linear constraints model *y*_*i**j*_ = *t*_*i*_*x*_*i**j*_:

23 $$\begin{array}{@{}rcl@{}} y_{ij} & \leq t_{i} \qquad \forall i\in \mathcal{I}, j\in \mathcal{J} \end{array}$$

24 $$\begin{array}{@{}rcl@{}} y_{ij} & \leq x_{ij} \qquad \forall i\in \mathcal{I}, j\in \mathcal{J} \end{array}$$

25 $$\begin{array}{@{}rcl@{}} (1-x_{ij})+y_{ij} & \geq t_{i} \qquad \forall i\in \mathcal{I}, j\in \mathcal{J} \end{array}$$

26 $$\begin{array}{@{}rcl@{}} y_{ij} , t_{i} & \geq 0 \qquad \forall i\in \mathcal{I}, j\in \mathcal{J} \end{array}$$

Therefore, constraint (2) in [S-1] can be replaced by constraints (21)-(26). Constraint (8) in [S-2] can be linearized identically.

Shirabe [28] describes flow-based contiguity constraints for districting problems. We adapt these constraints to model *receiving contiguity* and *sharing contiguity* in our neighborhood framework through equations (27)-(29) and (30)-(32), respectively. With receiving (sharing) contiguity, the suppliers (recipients) assigned to a recipient (supplier) form a continuous geography on the map. Let *m*_1_ (*m*_2_) be the maximum number of supply (demand) locations that can be assigned to a demand (supply) location. Parameter *a*_*i**k*_ = 1, if supply locations *i* and *k* are geographically adjacent, and 0 otherwise. We use flow variables $f_{ik}^{j}$ to model receiving contiguity and flow variables $g_{jk}^{i}$ to model sharing contiguity. Flow variable $f_{ik}^{j}$ denotes the flow from *i* to *k* (only defined when *a*_*i**k*_ = 1) destined for demand location *j*, while flow variable $g_{jk}^{i}$ denotes the flow from *j* to *k* (only defined when *a*_*j**k*_ = 1) destined for supply location *i*. The first three constraints involving the flow variables $f_{ik}^{j}$ ensure that if *x*_*i**j*_ = 1 for a supply location *i* and a demand location *j* that are non-adjacent, then every supply location on the path from *i* to *j* also supplies demand location *j*. The next set of three constraints involving the flow variables $g_{jk}^{i}$ ensure that if *x*_*i**j*_ = 1 for a supply location *i* and a demand location *j* that are non-adjacent, then every demand location on the path from *i* to *j* is also supplied by *i*.

27 $$\begin{array}{@{}rcl@{}} \sum\limits_{k=1}^{N_{sup}}f_{ik}^j a_{ik}-\sum\limits_{k=1}^{N_{sup}}f_{ki}^j a_{ki} & = & x_{ij} \quad\forall i \neq j, i\in \mathcal{I}, j\in \mathcal{J} \end{array}$$

28 $$\begin{array}{@{}rcl@{}} \sum\limits_{k=1}^{N_{sup}}f_{ki}^j a_{ki} & \leq & (m_1-1) x_{ij}\quad \forall i\in \mathcal{I},j\in \mathcal{J} \end{array}$$

29 $$\begin{array}{@{}rcl@{}} \sum\limits_{k=1}^{N_{sup}}f_{jk}^j a_{jk} & = & 0\quad \forall j\in \mathcal{J} \end{array}$$

30 $$\begin{array}{@{}rcl@{}} \sum\limits_{k=1}^{N_{dem}}g_{jk}^i a_{jk}-\sum\limits_{k=1}^{N_{dem}}g_{kj}^i a_{kj} & = & x_{ij} \quad\forall i \neq j, i\in \mathcal{I}, j\in \mathcal{J} \end{array}$$

31 $$\begin{array}{@{}rcl@{}} \sum\limits_{k=1}^{N_{dem}}g_{kj}^i a_{kj} & \leq & (m_2-1) x_{ij}\quad \forall i\in \mathcal{I}, j\in \mathcal{J} \end{array}$$

32 $$\begin{array}{@{}rcl@{}} \sum\limits_{k=1}^{N_{dem}}g_{ik}^i a_{ik} & = & 0\quad \forall i\in \mathcal{I} \end{array}$$

33 $$\begin{array}{@{}rcl@{}} f_{ik}^j & \geq & 0\quad \forall i,k \in \mathcal{I}, j\in \mathcal{J} \end{array}$$

34 $$\begin{array}{@{}rcl@{}} g_{jk}^i & \geq & 0\quad \forall i \in \mathcal{I}, k,j \in \mathcal{J} \end{array}$$

Together constraints (27)–(34) refer to constraint (6) in [S-1].

## Appendix C: Set partitioning model computational details

Table 8 contains the computational details of the set-partitioning model run on four-digit zip-clusters. We report the problem size, total number of cutting planes used by the solver, run time until termination, nodes explored, simplex iterations, best objective, best bound, and MIP gap. We observe that by two hours of running time, the MIP gap of [SP-1] and [SP-2] typically reaches below 0.02% and 0.12%, respectively, which corresponds to the difference in the s/d ratio at the fourth or higher decimal places between the best objective and best bound.

**Table 8:** Computational details of the set-partitioning model run on four-digit zip-clusters

*τ*_*m**a**x*_		Size		Cutting	Run time	Nodes	Simplex	Best	Best	MIP
(in NM)		Rows	Columns	Planes	(in secs)	explored	iterations	obj.	bound	gap
350	[SP-1]	1525	19061	2	7200	3444938	7290609	0.519	0.519	0.01%
	[SP-2]	1668	19062	22	6	1	2047	0.884	0.884	0.00%
400	[SP-1]	1527	24776	0	7200	2785686	9013065	0.537	0.537	0.01%
	[SP-2]	1670	24777	0	7200	262484	20797966	0.611	0.610	0.12%
450	[SP-1]	1528	30362	6	45	1	10922	0.543	0.543	0.01%
	[SP-2]	1671	30363	16	7200	72601	18971000	0.606	0.606	0.10%
500	[SP-1]	1528	35990	2420	7200	499437	6067640	0.551	0.551	0.02%
	[SP-2]	1671	35991	0	7200	85424	30217420	0.605	0.604	0.03%
550	[SP-1]	1528	42175	3005	7200	220154	6747918	0.554	0.554	0.01%
	[SP-2]	1671	42176	495	7200	44898	11247871	0.604	0.604	0.03%
600	[SP-1]	1528	48381	1936	7200	201431	1759417	0.555	0.555	0.01%
	[SP-2]	1671	48382	93	7200	26765	10007894	0.602	0.602	0.08%
650	[SP-1]	1528	54323	1908	7200	209106	2543837	0.554	0.554	0.01%
	[SP-2]	1671	54324	39	7200	28708	6409075	0.602	0.602	0.06%
700	[SP-1]	1528	59952	15	7200	837756	4521290	0.556	0.556	0.01%
	[SP-2]	1671	59953	20	7200	22964	5031591	0.602	0.601	0.05%

## Appendix D: DSA neighborhoods

Tables 9, 10, and 11, present the neighborhoods obtained by our model based on the DSA version when the maximum distance to any DSA in the neighborhood is constrained to 500 NM, 600 NM, and 700 NM, respectively. The column “Radius” provides the radius (in terms of the transplant volume-weighted distance, as discussed in Section 4.2) of each neighborhood. The column “Neighbors” contains the DSAs with which the DSA in the first column will share its organs.

**Table 9:** Geographical allocation policy for DSAs when the maximum permitted distance to a neighboring DSA is 500 NM

DSA	Radius	Neighbors
	(in NM)	
ALOB	336	KYDA, ALOB, NCCM, TNDS, MSOP, AROR, SCOP, TNMS, FLUF, GALL, LAOP
AROR	305	MSOP, AROR, TXSB, TNMS, MOMA, MWOB, TXGC, LAOP, OKOP
AZOB	499	AZOB, CORS, UTOP, CASD, CAOP
CADN	366	HIOP, CADN, CASD, CAOP
CAGS	318	HIOP, CADN, CAOP
CAOP	499	HIOP, AZOB, UTOP, CADN, CASD, CAOP
CASD	366	HIOP, AZOB, CADN, CASD, CAOP
CORS	316	CORS, UTOP
CTOP	203	MAOB, NYFL, CTOP, PADV, NJTO, NYRT
DCTC	339	OHOV, NCNC, MAOB, NYFL, PATF, NCCM, CTOP, DCTC, PADV, VATB, MDPC, OHLP, NJTO, OHLB, NYRT
FLFH	492	NCNC, ALOB, PRLL, NCCM, TNDS, FLMP, MSOP, SCOP, FLFH, FLUF, FLWC, GALL
FLMP	472	PRLL, FLMP, SCOP, FLFH, FLUF, FLWC, GALL
FLUF	422	NCNC, ALOB, PRLL, NCCM, TNDS, FLMP, MSOP, SCOP, FLFH, FLUF, FLWC, GALL, LAOP
FLWC	488	NCNC, ALOB, PRLL, NCCM, FLMP, MSOP, SCOP, FLFH, FLUF, FLWC, GALL
GALL	484	OHOV, NCNC, KYDA, ALOB, PATF, NCCM, DCTC, TNDS, FLMP, MSOP, AROR, SCOP, TNMS, VATB, MOMA, OHLP, FLFH, OHLB, FLUF, INOP, FLWC, GALL, LAOP
HIOP	NA	HIOP, CADN, CASD, CAOP
IAOP	210	MNOP, WIUW, IAOP, NEOR
ILIP	447	OHOV, MNOP, KYDA, PATF, TNDS, WIUW, ILIP, AROR, IAOP, TNMS, MOMA, MWOB, OHLP, WIDN, NEOR, OHLB, INOP, MIOP
INOP	224	OHOV, KYDA, ILIP, OHLP, WIDN, OHLB, INOP, MIOP
KYDA	497	OHOV, NCNC, KYDA, ALOB, NYFL, PATF, NCCM, DCTC, TNDS, PADV, WIUW, ILIP, MSOP, AROR, IAOP, SCOP, TNMS, VATB, MDPC, MOMA, MWOB, OHLP, WIDN, OHLB, FLUF, INOP, GALL, MIOP
LAOP	396	TXSA, ALOB, MSOP, AROR, TXSB, TNMS, TXGC, LAOP, OKOP
MAOB	170	MAOB, CTOP, NJTO, NYRT
MDPC	308	NCNC, MAOB, NYFL, PATF, CTOP, DCTC, PADV, VATB, MDPC, OHLP, NJTO, OHLB, NYRT
MIOP	482	OHOV, MNOP, KYDA, NYFL, PATF, NCCM, DCTC, TNDS, PADV, WIUW, ILIP, IAOP, VATB, MDPC, MOMA, OHLP, NJTO, WIDN, OHLB, INOP, NYRT, MIOP
MNOP	270	MNOP, WIUW, IAOP, NEOR
MOMA	233	ILIP, AROR, TNMS, MOMA, MWOB
MSOP	406	ALOB, TNDS, MSOP, AROR, TXSB, TNMS, MOMA, TXGC, FLUF, GALL, LAOP
MWOB	477	MNOP, KYDA, WIUW, ILIP, AROR, IAOP, TXSB, TNMS, MOMA, MWOB, WIDN, CORS, NEOR, TXGC, INOP, OKOP
NCCM	403	OHOV, NCNC, KYDA, ALOB, PATF, NCCM, DCTC, TNDS, PADV, SCOP, VATB, MDPC, OHLP, FLFH, OHLB, FLUF, INOP, GALL
NCNC	489	OHOV, NCNC, KYDA, ALOB, NYFL, PATF, NCCM, CTOP, DCTC, TNDS, PADV, SCOP, VATB, MDPC, OHLP, NJTO, FLFH, OHLB, FLUF, INOP, FLWC, NYRT, GALL, MIOP
NEOR	390	MNOP, WIUW, IAOP, MOMA, MWOB, CORS, NEOR, OKOP
NJTO	188	MAOB, NYFL, CTOP, DCTC, PADV, MDPC, NJTO, NYRT
NMOP	282	AZOB
NVLV	308	AZOB, CADN, CASD, CAOP
NYAP	182	MAOB, NYFL, CTOP, PADV, NJTO, NYRT
NYFL	458	OHOV, MAOB, NYFL, PATF, CTOP, DCTC, PADV, VATB, MDPC, OHLP, NJTO, OHLB, INOP, NYRT, MIOP
NYRT	193	MAOB, NYFL, CTOP, PADV, MDPC, NJTO, NYRT
NYWN	234	NYFL, PATF, PADV, OHLB, MIOP
OHLB	459	OHOV, NCNC, KYDA, MAOB, NYFL, PATF, NCCM, CTOP, DCTC, TNDS, PADV, WIUW, ILIP, SCOP, VATB, MDPC, MOMA, OHLP, NJTO, WIDN, OHLB, INOP, NYRT, MIOP
OHLC	329	OHOV, KYDA, PATF, DCTC, TNDS, WIUW, ILIP, OHLP, WIDN, OHLB, INOP, MIOP
OHLP	307	OHOV, KYDA, PATF, NCCM, DCTC, TNDS, ILIP, VATB, MDPC, OHLP, WIDN, OHLB, INOP, MIOP
OHOV	284	OHOV, KYDA, PATF, NCCM, TNDS, ILIP, OHLP, WIDN, OHLB, INOP, MIOP
OKOP	475	TXSA, MSOP, AROR, IAOP, TXSB, TNMS, MOMA, MWOB, CORS, NEOR, TXGC, LAOP, OKOP
ORUO	234	WALC, HIOP, ORUO
PADV	235	MAOB, NYFL, PATF, CTOP, DCTC, PADV, VATB, MDPC, NJTO, NYRT
PATF	278	OHOV, NCNC, NYFL, PATF, DCTC, PADV, VATB, MDPC, OHLP, NJTO, OHLB, NYRT, MIOP
PRLL	NA	PRLL, FLMP, FLWC
SCOP	326	NCNC, KYDA, ALOB, NCCM, TNDS, SCOP, VATB, FLFH, FLUF, GALL
TNDS	493	OHOV, NCNC, KYDA, ALOB, PATF, NCCM, DCTC, TNDS, ILIP, MSOP, AROR, SCOP, TNMS, VATB, MDPC, MOMA, MWOB, OHLP, WIDN, FLFH, OHLB, FLUF, INOP, GALL, MIOP, LAOP
TNMS	492	OHOV, KYDA, ALOB, NCCM, TNDS, ILIP, MSOP, AROR, IAOP, SCOP, TXSB, TNMS, MOMA, MWOB, OHLP, WIDN, TXGC, FLUF, INOP, GALL, LAOP, OKOP
TXGC	276	TXSA, TXSB, TXGC, LAOP, OKOP
TXSA	363	TXSA, TXSB, TXGC, LAOP
TXSB	218	TXSA, TXSB, TXGC, OKOP
UTOP	480	AZOB, CORS, UTOP, CADN
VATB	196	NCNC, DCTC, PADV, VATB, MDPC
WALC	234	WALC, ORUO
WIDN	284	OHOV, WIUW, ILIP, IAOP, WIDN, INOP, MIOP
WIUW	426	OHOV, MNOP, WIUW, ILIP, IAOP, MOMA, MWOB, OHLP, WIDN, NEOR, OHLB, INOP, MIOP

**Table 10:** Geographical allocation policy for DSAs when the maximum permitted distance to a neighboring DSA is 600 NM

DSA	Radius	Neighbors
	(in NM)	
ALOB	517	OHOV, NCNC, KYDA, ALOB, NCCM, TNDS, MSOP, AROR, SCOP, TNMS, VATB, MOMA, OHLP, FLFH, TXGC, FLUF, INOP, FLWC, GALL, LAOP
AROR	569	OHOV, TXSA, KYDA, ALOB, TNDS, ILIP, MSOP, AROR, IAOP, TXSB, TNMS, MOMA, MWOB, WIDN, NEOR, TXGC, FLUF, INOP, GALL, LAOP, OKOP
AZOB	546	AZOB, CORS, UTOP, CADN, CASD, CAOP
CADN	366	HIOP, CADN, CASD, CAOP
CAGS	446	HIOP, ORUO, UTOP, CADN, CASD, CAOP
CAOP	499	HIOP, AZOB, UTOP, CADN, CASD, CAOP
CASD	265	HIOP, AZOB, CASD, CAOP
CORS	316	CORS, UTOP
CTOP	244	MAOB, NYFL, CTOP, PADV, MDPC, NJTO, NYRT
DCTC	346	OHOV, NCNC, MAOB, NYFL, PATF, NCCM, CTOP, DCTC, PADV, SCOP, VATB, MDPC, OHLP, NJTO, OHLB, NYRT
FLFH	578	NCNC, ALOB, PRLL, NCCM, TNDS, FLMP, MSOP, SCOP, TNMS, VATB, FLFH, FLUF, FLWC, GALL, LAOP
FLMP	573	NCNC, ALOB, PRLL, NCCM, FLMP, SCOP, FLFH, FLUF, FLWC, GALL
FLUF	592	OHOV, NCNC, KYDA, ALOB, PRLL, NCCM, TNDS, FLMP, MSOP, AROR, SCOP, TNMS, VATB, OHLP, FLFH, FLUF, FLWC, GALL, LAOP
FLWC	330	PRLL, FLMP, FLFH, FLUF, FLWC, GALL
GALL	565	OHOV, NCNC, KYDA, ALOB, PATF, NCCM, DCTC, TNDS, PADV, FLMP, ILIP, MSOP, AROR, SCOP, TNMS, VATB, MDPC, MOMA, OHLP, FLFH, OHLB, FLUF, INOP, FLWC, GALL, MIOP, LAOP
HIOP	NA	HIOP, CADN, CASD, CAOP
IAOP	335	MNOP, WIUW, ILIP, IAOP, MOMA, MWOB, WIDN, NEOR, INOP
ILIP	365	OHOV, MNOP, KYDA, TNDS, WIUW, ILIP, IAOP, MOMA, MWOB, OHLP, WIDN, OHLB, INOP, MIOP
INOP	571	OHOV, MNOP, NCNC, KYDA, ALOB, NYFL, PATF, NCCM, DCTC, TNDS, PADV, WIUW, ILIP, MSOP, AROR, IAOP, SCOP, TNMS, VATB, MDPC, MOMA, MWOB, OHLP, NJTO, WIDN, NEOR, OHLB, INOP, NYRT, GALL, MIOP, OKOP
KYDA	497	OHOV, NCNC, KYDA, ALOB, NYFL, PATF, NCCM, DCTC, TNDS, PADV, WIUW, ILIP, MSOP, AROR, IAOP, SCOP, TNMS, VATB, MDPC, MOMA, MWOB, OHLP, WIDN, OHLB, FLUF, INOP, GALL, MIOP
LAOP	396	TXSA, ALOB, MSOP, AROR, TXSB, TNMS, TXGC, LAOP, OKOP
MAOB	170	MAOB, CTOP, NJTO, NYRT
MDPC	407	OHOV, NCNC, KYDA, MAOB, NYFL, PATF, NCCM, CTOP, DCTC, PADV, SCOP, VATB, MDPC, OHLP, NJTO, OHLB, NYRT, MIOP
MIOP	594	OHOV, MNOP, NCNC, KYDA, MAOB, NYFL, PATF, NCCM, CTOP, DCTC, TNDS, PADV, WIUW, ILIP, IAOP, SCOP, TNMS, VATB, MDPC, MOMA, MWOB, OHLP, NJTO, WIDN, NEOR, OHLB, INOP, NYRT, GALL, MIOP
MNOP	270	MNOP, WIUW, IAOP, NEOR
MOMA	600	OHOV, MNOP, NCNC, KYDA, ALOB, PATF, NCCM, TNDS, WIUW, ILIP, MSOP, AROR, IAOP, SCOP, TXSB, TNMS, MOMA, MWOB, OHLP, WIDN, NEOR, OHLB, TXGC, FLUF, INOP, GALL, MIOP, LAOP, OKOP
MSOP	482	TXSA, KYDA, ALOB, NCCM, TNDS, MSOP, AROR, SCOP, TXSB, TNMS, MOMA, MWOB, FLFH, TXGC, FLUF, FLWC, GALL, LAOP, OKOP
MWOB	590	OHOV, TXSA, MNOP, KYDA, ALOB, TNDS, WIUW, ILIP, MSOP, AROR, IAOP, TXSB, TNMS, MOMA, MWOB, WIDN, CORS, NEOR, TXGC, INOP, MIOP, LAOP, OKOP
NCCM	498	OHOV, NCNC, KYDA, ALOB, PATF, NCCM, DCTC, TNDS, PADV, ILIP, MSOP, SCOP, TNMS, VATB, MDPC, MOMA, OHLP, NJTO, FLFH, OHLB, FLUF, INOP, FLWC, NYRT, GALL, MIOP
NCNC	570	OHOV, NCNC, KYDA, ALOB, MAOB, NYFL, PATF, NCCM, CTOP, DCTC, TNDS, PADV, ILIP, MSOP, SCOP, TNMS, VATB, MDPC, OHLP, NJTO, FLFH, OHLB, FLUF, INOP, FLWC, NYRT, GALL, MIOP
NEOR	377	MNOP, IAOP, MOMA, MWOB, CORS, NEOR, OKOP
NJTO	254	MAOB, NYFL, PATF, CTOP, DCTC, PADV, VATB, MDPC, NJTO, NYRT
NMOP	282	AZOB
NVLV	308	AZOB, CADN, CASD, CAOP
NYAP	182	MAOB, NYFL, CTOP, PADV, NJTO, NYRT
NYFL	582	OHOV, NCNC, KYDA, MAOB, NYFL, PATF, NCCM, CTOP, DCTC, PADV, WIUW, ILIP, SCOP, VATB, MDPC, OHLP, NJTO, WIDN, OHLB, INOP, NYRT, MIOP
NYRT	195	MAOB, NYFL, CTOP, DCTC, PADV, MDPC, NJTO, NYRT
NYWN	326	MAOB, NYFL, PATF, CTOP, DCTC, PADV, MDPC, OHLP, NJTO, OHLB, NYRT, MIOP
OHLB	457	OHOV, NCNC, KYDA, MAOB, NYFL, PATF, NCCM, CTOP, DCTC, TNDS, PADV, WIUW, ILIP, SCOP, VATB, MDPC, OHLP, NJTO, WIDN, OHLB, INOP, NYRT, MIOP
OHLC	346	OHOV, KYDA, PATF, NCCM, DCTC, TNDS, WIUW, ILIP, MDPC, MOMA, OHLP, WIDN, OHLB, INOP, MIOP
OHLP	366	OHOV, NCNC, KYDA, NYFL, PATF, NCCM, DCTC, TNDS, PADV, ILIP, SCOP, VATB, MDPC, OHLP, WIDN, OHLB, INOP, MIOP
OHOV	599	OHOV, MNOP, NCNC, KYDA, ALOB, NYFL, PATF, NCCM, CTOP, DCTC, TNDS, PADV, WIUW, ILIP, MSOP, AROR, IAOP, SCOP, TNMS, VATB, MDPC, MOMA, MWOB, OHLP, NJTO, WIDN, NEOR, OHLB, FLUF, INOP, NYRT, GALL, MIOP
OKOP	397	TXSA, AROR, TXSB, TNMS, MOMA, MWOB, NEOR, TXGC, LAOP, OKOP
ORUO	234	WALC, HIOP, ORUO
PADV	275	MAOB, NYFL, PATF, CTOP, DCTC, PADV, VATB, MDPC, NJTO, OHLB, NYRT
PATF	406	OHOV, NCNC, KYDA, MAOB, NYFL, PATF, NCCM, CTOP, DCTC, TNDS, PADV, ILIP, SCOP, VATB, MDPC, OHLP, NJTO, WIDN, OHLB, INOP, NYRT, MIOP
PRLL	NA	PRLL, FLMP
SCOP	450	OHOV, NCNC, KYDA, ALOB, PATF, NCCM, DCTC, TNDS, PADV, SCOP, TNMS, VATB, MDPC, OHLP, FLFH, OHLB, FLUF, INOP, FLWC, GALL
TNDS	525	OHOV, NCNC, KYDA, ALOB, PATF, NCCM, DCTC, TNDS, PADV, ILIP, MSOP, AROR, IAOP, SCOP, TNMS, VATB, MDPC, MOMA, MWOB, OHLP, WIDN, FLFH, OHLB, FLUF, INOP, FLWC, GALL, MIOP, LAOP
TNMS	594	OHOV, TXSA, NCNC, KYDA, ALOB, PATF, NCCM, TNDS, WIUW, ILIP, MSOP, AROR, IAOP, SCOP, TXSB, TNMS, VATB, MOMA, MWOB, OHLP, WIDN, NEOR, FLFH, OHLB, TXGC, FLUF, INOP, FLWC, GALL, MIOP, LAOP, OKOP
TXGC	276	TXSA, TXSB, TXGC, LAOP, OKOP
TXSA	179	TXSA, TXSB, TXGC
TXSB	598	TXSA, ALOB, MSOP, AROR, TXSB, TNMS, MOMA, MWOB, CORS, NEOR, TXGC, LAOP, OKOP
UTOP	480	AZOB, CORS, UTOP, CADN
VATB	437	OHOV, NCNC, KYDA, MAOB, NYFL, PATF, NCCM, CTOP, DCTC, TNDS, PADV, SCOP, VATB, MDPC, OHLP, NJTO, OHLB, INOP, NYRT, GALL, MIOP
WALC	234	WALC, ORUO
WIDN	200	WIUW, ILIP, WIDN, INOP, MIOP
WIUW	248	MNOP, WIUW, ILIP, IAOP, WIDN, MIOP

The radii for each zip-code cluster in the zip-code version (which is too large to include as a table) can be obtained by contacting the authors.

**Table 11:** Geographical allocation policy for DSAs when the maximum permitted distance to a neighboring DSA is 700 NM

DSA	Radius	Neighbors
	(in NM)	
ALOB	684	OHOV, TXSA, NCNC, KYDA, ALOB, PATF, NCCM, DCTC, TNDS, PADV, FLMP, ILIP, MSOP, AROR, IAOP, SCOP, TXSB, TNMS, VATB, MDPC, MOMA, MWOB, OHLP, WIDN, FLFH, OHLB, TXGC, FLUF, INOP, FLWC, GALL, MIOP, LAOP, OKOP
AROR	297	MSOP, AROR, TXSB, TNMS, MOMA, MWOB, LAOP, OKOP
AZOB	546	AZOB, CORS, UTOP, CADN, CASD, CAOP
CADN	655	WALC, HIOP, AZOB, ORUO, UTOP, CADN, CASD, CAOP
CAGS	318	HIOP, CADN, CAOP
CAOP	316	HIOP, AZOB, CADN, CASD, CAOP
CASD	537	HIOP, AZOB, UTOP, CADN, CASD, CAOP
CORS	316	CORS, UTOP
CTOP	343	MAOB, NYFL, PATF, CTOP, DCTC, PADV, MDPC, NJTO, NYRT
DCTC	421	OHOV, NCNC, KYDA, MAOB, NYFL, PATF, NCCM, CTOP, DCTC, PADV, SCOP, VATB, MDPC, OHLP, NJTO, OHLB, INOP, NYRT, MIOP
FLFH	687	OHOV, NCNC, KYDA, ALOB, PRLL, NCCM, DCTC, TNDS, FLMP, MSOP, SCOP, TNMS, VATB, MDPC, OHLP, FLFH, FLUF, FLWC, GALL, LAOP
FLMP	287	PRLL, FLMP, FLFH, FLUF, FLWC
FLUF	674	OHOV, NCNC, KYDA, ALOB, PATF, PRLL, NCCM, DCTC, TNDS, FLMP, MSOP, AROR, SCOP, TNMS, VATB, MDPC, MOMA, OHLP, FLFH, OHLB, FLUF, INOP, FLWC, GALL, LAOP
FLWC	488	NCNC, ALOB, PRLL, NCCM, FLMP, MSOP, SCOP, FLFH, FLUF, FLWC, GALL
GALL	671	OHOV, NCNC, KYDA, ALOB, NYFL, PATF, NCCM, DCTC, TNDS, PADV, FLMP, ILIP, MSOP, AROR, SCOP, TNMS, VATB, MDPC, MOMA, MWOB, OHLP, NJTO, WIDN, FLFH, OHLB, TXGC, FLUF, INOP, FLWC, NYRT, GALL, MIOP, LAOP, OKOP
HIOP	NA	HIOP, CASD
IAOP	235	MNOP, WIUW, ILIP, IAOP, MWOB, WIDN, NEOR
ILIP	447	OHOV, MNOP, KYDA, PATF, TNDS, WIUW, ILIP, AROR, IAOP, TNMS, MOMA, MWOB, OHLP, WIDN, NEOR, OHLB, INOP, MIOP
INOP	510	OHOV, MNOP, NCNC, KYDA, ALOB, NYFL, PATF, NCCM, DCTC, TNDS, PADV, WIUW, ILIP, MSOP, AROR, IAOP, SCOP, TNMS, VATB, MDPC, MOMA, MWOB, OHLP, WIDN, NEOR, OHLB, INOP, GALL, MIOP
KYDA	696	OHOV, MNOP, NCNC, KYDA, ALOB, MAOB, NYFL, PATF, NCCM, CTOP, DCTC, TNDS, PADV, WIUW, ILIP, MSOP, AROR, IAOP, SCOP, TNMS, VATB, MDPC, MOMA, MWOB, OHLP, NJTO, WIDN, NEOR, FLFH, OHLB, TXGC, FLUF, INOP, FLWC, NYRT, GALL, MIOP, LAOP, OKOP
LAOP	396	TXSA, ALOB, MSOP, AROR, TXSB, TNMS, TXGC, LAOP, OKOP
MAOB	170	MAOB, CTOP, NJTO, NYRT
MDPC	645	OHOV, NCNC, KYDA, ALOB, MAOB, NYFL, PATF, NCCM, CTOP, DCTC, TNDS, PADV, WIUW, ILIP, SCOP, VATB, MDPC, OHLP, NJTO, WIDN, OHLB, FLUF, INOP, NYRT, GALL, MIOP
MIOP	216	OHOV, ILIP, OHLP, WIDN, OHLB, INOP, MIOP
MNOP	270	MNOP, WIUW, IAOP, NEOR
MOMA	694	OHOV, TXSA, MNOP, NCNC, KYDA, ALOB, PATF, NCCM, DCTC, TNDS, WIUW, ILIP, MSOP, AROR, IAOP, SCOP, TXSB, TNMS, VATB, MDPC, MOMA, MWOB, OHLP, WIDN, CORS, NEOR, OHLB, TXGC, FLUF, INOP, GALL, MIOP, LAOP, OKOP
MSOP	418	KYDA, ALOB, TNDS, MSOP, AROR, TXSB, TNMS, MOMA, TXGC, FLUF, GALL, LAOP, OKOP
MWOB	590	OHOV, TXSA, MNOP, KYDA, ALOB, TNDS, WIUW, ILIP, MSOP, AROR, IAOP, TXSB, TNMS, MOMA, MWOB, WIDN, CORS, NEOR, TXGC, INOP, MIOP, LAOP, OKOP
NCCM	238	NCNC, KYDA, NCCM, TNDS, SCOP, VATB, GALL
NCNC	320	NCNC, KYDA, PATF, NCCM, DCTC, TNDS, PADV, SCOP, VATB, MDPC, OHLP, GALL
NEOR	377	MNOP, IAOP, MOMA, MWOB, CORS, NEOR, OKOP
NJTO	254	MAOB, NYFL, PATF, CTOP, DCTC, PADV, MDPC, NJTO, NYRT
NMOP	282	AZOB
NVLV	311	AZOB, UTOP, CADN, CASD, CAOP
NYAP	182	MAOB, NYFL, CTOP, PADV, NJTO, NYRT
NYFL	186	NYFL, PADV
NYRT	195	MAOB, NYFL, CTOP, DCTC, PADV, MDPC, NJTO, NYRT
NYWN	153	NYFL, OHLB
OHLB	484	OHOV, NCNC, KYDA, MAOB, NYFL, PATF, NCCM, CTOP, DCTC, TNDS, PADV, WIUW, ILIP, SCOP, VATB, MDPC, MOMA, OHLP, NJTO, WIDN, OHLB, INOP, NYRT, GALL, MIOP
OHLC	678	OHOV, MNOP, NCNC, KYDA, ALOB, MAOB, NYFL, PATF, NCCM, CTOP, DCTC, TNDS, PADV, WIUW, ILIP, MSOP, AROR, IAOP, SCOP, TNMS, VATB, MDPC, MOMA, MWOB, OHLP, NJTO, WIDN, NEOR, OHLB, FLUF, INOP, NYRT, GALL, MIOP, OKOP
OHLP	624	OHOV, MNOP, NCNC, KYDA, ALOB, MAOB, NYFL, PATF, NCCM, CTOP, DCTC, TNDS, PADV, WIUW, ILIP, MSOP, AROR, IAOP, SCOP, TNMS, VATB, MDPC, MOMA, MWOB, OHLP, NJTO, WIDN, OHLB, FLUF, INOP, NYRT, GALL, MIOP
OHOV	410	OHOV, NCNC, KYDA, ALOB, PATF, NCCM, DCTC, TNDS, PADV, WIUW, ILIP, SCOP, TNMS, VATB, MDPC, MOMA, OHLP, WIDN, OHLB, INOP, GALL, MIOP
OKOP	418	TXSA, MSOP, AROR, TXSB, TNMS, MOMA, MWOB, NEOR, TXGC, LAOP, OKOP
ORUO	662	WALC, HIOP, ORUO, UTOP, CADN, CAOP
PADV	235	MAOB, NYFL, PATF, CTOP, DCTC, PADV, VATB, MDPC, NJTO, NYRT
PATF	544	OHOV, NCNC, KYDA, ALOB, MAOB, NYFL, PATF, NCCM, CTOP, DCTC, TNDS, PADV, WIUW, ILIP, SCOP, VATB, MDPC, MOMA, OHLP, NJTO, WIDN, OHLB, INOP, NYRT, GALL, MIOP
PRLL	NA	PRLL, FLMP, FLFH, FLWC
SCOP	263	NCNC, NCCM, TNDS, SCOP, VATB, FLUF, GALL
TNDS	694	OHOV, NCNC, KYDA, ALOB, NYFL, PATF, NCCM, CTOP, DCTC, TNDS, PADV, WIUW, FLMP, ILIP, MSOP, AROR, IAOP, SCOP, TXSB, TNMS, VATB, MDPC, MOMA, MWOB, OHLP, NJTO, WIDN, NEOR, FLFH, OHLB, TXGC, FLUF, INOP, FLWC, NYRT, GALL, MIOP, LAOP, OKOP
TNMS	561	OHOV, TXSA, NCNC, KYDA, ALOB, NCCM, TNDS, WIUW, ILIP, MSOP, AROR, IAOP, SCOP, TXSB, TNMS, MOMA, MWOB, OHLP, WIDN, NEOR, OHLB, TXGC, FLUF, INOP, GALL, MIOP, LAOP, OKOP
TXGC	276	TXSA, TXSB, TXGC, LAOP, OKOP
TXSA	179	TXSA, TXSB, TXGC
TXSB	598	TXSA, ALOB, MSOP, AROR, TXSB, TNMS, MOMA, MWOB, CORS, NEOR, TXGC, LAOP, OKOP
UTOP	537	AZOB, ORUO, CORS, UTOP, CADN, CASD, CAOP
VATB	517	OHOV, NCNC, KYDA, ALOB, MAOB, NYFL, PATF, NCCM, CTOP, DCTC, TNDS, PADV, SCOP, VATB, MDPC, OHLP, NJTO, OHLB, FLUF, INOP, NYRT, GALL, MIOP
WALC	234	WALC, ORUO
WIDN	423	OHOV, MNOP, KYDA, PATF, WIUW, ILIP, IAOP, MOMA, MWOB, OHLP, WIDN, OHLB, INOP, MIOP
WIUW	248	MNOP, WIUW, ILIP, IAOP, WIDN, MIOP

## Footnotes

1 https://www.srtr.org/reports-tools/waiting-list-calculator/, accessed June 26, 2020.

2 This is seen for example in 2016 data [22] where the highest MMaT is 39 and the lowest MMaT is 20.

3 Miriam Holman v. HHS, (S.D.N.Y 17-CV-09041).

4 Cruz et al. v. HHS, (S.D.N.Y 18-CV-06371).

5 https://optn.transplant.hrsa.gov/media/2506/geography_recommendations_report_201806.pdf, accessed April 30, 2022.

6 In a fixed radius circle policy, the radii of the circles around the donor hospitals are identical, whereas in a heterogeneous radii circle policy, the radii of circles around the donor hospital can take different values. We drop radius/radii and refer to them as a fixed circle and a heterogeneous circle policy.

7 https://unos.org/policy/liver-distribution/, accessed December 1, 2021.

8 Deceased-donor livers vary in quality, and marginal livers are more likely to be used and less likely to be discarded when more competition exists among transplant centers [12, 16]. Thus UNOS requires that a minimum number of transplant centers be in contention for organs from a supply location.

9 We consider incident patients so that the model parameters are not biased due to accumulated disparity, and thus are exogenous to the geographical scheme.

10 For every DSA with demand, there are close to three (142/52 = 2.73) transplant centers.

11 Recall that the optimization variable is *x*_*i**r*_. In the zip-code cluster version, *i* is the three-digit (four-digit) zip-code cluster, and *r* is the radius selected (from the discrete set of possible radii choices).

12 To represent a DSA on the map, we averaged the latitude and longitude values of the transplant centers in that DSA.
